# Systematics of the *Calotes irawadi* complex (Squamata, Agamidae) with two newly described species from Thailand

**DOI:** 10.3897/zookeys.1281.175455

**Published:** 2026-06-03

**Authors:** Arpapan Prakobkarn, George R. Zug, Nontivich Tandavanitj, Thongchai Ngamprasertwong

**Affiliations:** 1 Ph.D. Program in Zoology, Department of Biology, Faculty of Science, Chulalongkorn University, Bangkok 10330, Thailand National Museum of Natural History, Smithsonian Institution Washington United States of America; 2 Department of Vertebrate Zoology, National Museum of Natural History, Smithsonian Institution, Washington, DC 20560, USA Ph.D. Program in Zoology, Department of Biology, Faculty of Science, Chulalongkorn University Bangkok Thailand; 3 Department of Biology, Faculty of Science, Chulalongkorn University, Bangkok 10330, Thailand Department of Biology, Faculty of Science, Chulalongkorn University Bangkok Thailand

**Keywords:** Morphometric analysis, osteology, phylogenetic analysis, Southeast Asia, taxonomy

## Abstract

Two new species of *Calotes* lizards, *C. thailandensis***sp. nov**. and *C. maehongsonensis***sp. nov**., are diagnosed and described from Thailand. These new species are most closely related to *C. irawadi* and *C. wangi*, which are members of the *C. irawadi* complex, supported by phylogenetic analysis of mitochondrial DNA data (ND2 and COI genes) coupled with morphometric and osteological data. *Calotes thailandensis***sp. nov**. and *C. maehongsonensis***sp. nov**. are distinguished from true *C. irawadi* by having a wider PelvW, but a smaller head size in adult males. In particular, adult males of *C. thailandensis***sp. nov**. obviously differ from both *C. irawadi* and *C. wangi* by having a longer supratympanic spine, whereas *C. maehongsonensis***sp. nov**. has a distinctly longer hindlimb than that of *C. irawadi* and *C. wangi*. As a result, two new species increase the list of known *Calotes* species in Thailand to four species; *C. thailandensis***sp. nov**., *C. maehongsonensis***sp. nov**., *C. emma* and *C. goetzi*.

## Introduction

The agamid lizards (Squamata: Agamidae) of the genus *Calotes* Cuvier, 1817 currently include 33 nominal species (Uetz et al. 2025). In the past, *Calotes cf. versicolor* was a species with the widest distribution from South Asia to Southeast Asia, including parts of East Asia (Smith 1935; Auffenberg and Rehman 1993, 1995; Chan-ard et al. 2015; Huang et al. 2023). On several occasions, noticeable morphological variation was reported. Zug et al. (2006) revealed that morphological characters of *C. cf. versicolor* from Myanmar deviated from the original description. Based on the analyses of ND2 sequences, *C. cf. versicolor* from some localities in central Myanmar was subsequently described as *C. irawadi* Zug, Brown, Schulte & Vindum, 2006 based on its phylogenetic position in a clade separated from true *C. versicolor* from India. Currently, its distribution extends across northeastern India (Assam, Arunachal Pradesh, Tripura, Manipur, and Mizoram) to Myanmar and western Yunnan, China (Zug et al. 2006; Das et al. 2009; Gowande et al. 2021; Liu et al. 2021; Tariang et al. 2022; Decemson et al. 2023).

Gowande et al. (2021) revealed that *C. cf. versicolor* from northeastern India, Vietnam, Cambodia, and far eastern China were grouped with *C. irawadi* from Myanmar based on COI and 16S sequences, indicating the existence of a *C. irawadi* complex in Southeast Asia. The possible existence of cryptic species has been suggested (Gowande et al. 2021; Tantrawatpan et al. 2021; Prakobkarn et al. 2024) and *C. wangi* was recently described from *C. cf. versicolor* in southern China and northern Vietnam by Huang et al. (2023). Zug et al. (2006) described two *Calotes* species, *C. irawadi* and *C. htunwini*, from Chatthin in the central dry zone of Myanmar and also indicated that certain populations in Myanmar were morphologically distinct compared to *C. irawadi* and *C. htunwini*, particularly those from Moyingyi (in Bago, southern Myanmar) as well as Thailand. Their taxonomic status remained ambiguous. Unlike *C. htunwini*, this group shares vertical and obliquely upward oriented scales on side of neck and shoulders, whereas *C. irawadi* differs from others by having a small supratympanic spine (0.5 or less the tympanum diameter) and a middorsal crest equal to tympanum diameter, and fewer middorsal scales (mean 49) (Zug et al. 2006). Moreover, the Thai population has a moderately developed supratympanic spine (half or less the maximum tympanum diameter), and the presence of dorsolateral stripes (DorsalSt) and nuchal spots (NucSpot) is distinct from *C. irawadi* (Zug et al. 2006). From 2008–2010, during field surveys in northern and southern Thailand, morphological variation among those populations was also observed (Prakobkarn et al. 2016).

Furthermore, morphological evidence and phylogenetic analyses also show genetic variation within the species complex. Previous molecular studies have demonstrated the presence of several subclades nested within the *C. irawadi* clade with substantial genetic distances between subclades (Zug et al. 2006; Gowande et al. 2021; Tantrawatpan et al. 2021; Decemson et al. 2023). Notably within *C. irawadi*, populations were assigned to several divergent lineages based on the mitochondrial COI gene, some of which occurred on both banks of the Mekong River in Thailand and Laos (Tantrawatpan et al. 2021). To date, morphological and molecular analyses of *C. irawadi* throughout Thailand have confirmed the presence of more than one species embedded within the Thai *C. irawadi* complex (Prakobkarn et al. 2016; Prakobkarn et al. 2024). Therefore, this study aims to clarify the taxonomic status of these populations. We hypothesize there are at least two new species within this species group.

## Materials and methods

**Figure 1.**
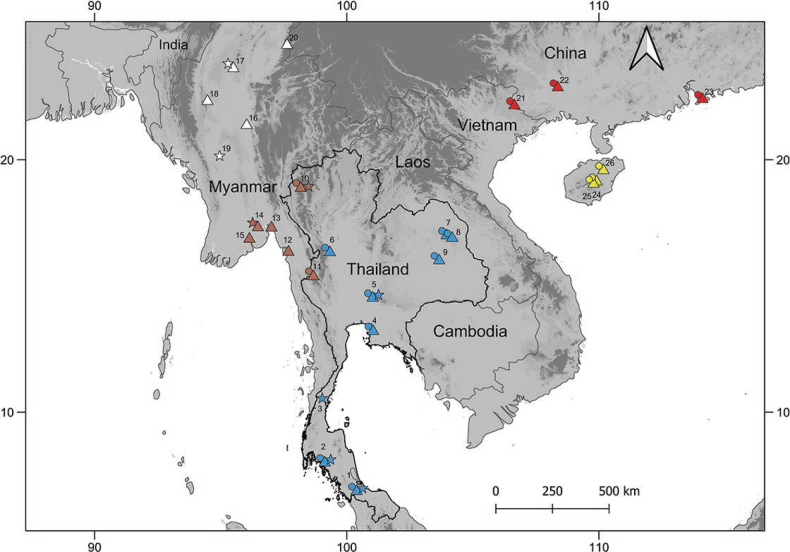
Sample locality map of *Calotes thailandensis* sp. nov. (blue) (1–9), *C. maehongsonensis* sp. nov. (brown) (10–15), *C. irawadi* (white) (16–20), *C. wangi wangi* (21–23) (red), and *C. wangi hainanensis* (24–26) (yellow) from Thailand and nearby countries. Symbols correspond to molecular (triangle for ND2; circle for COI genes) and morphological (star) analyses. Localities include Thailand: Songkhla (type locality of *C. thailandensis* sp. nov.) (1), Krabi (2), Ranong (3), Chon Buri (4), Saraburi (5), Kamphaeng Phet (6), Sakon Nakhon (7), Mukdahan (8), Roi Et (9), Mae Hong Son (type locality of *C. maehongsonensis* sp. nov.) (10), and Kanchanaburi (11); Myanmar: Mon, Mawlamyine (12), Mon, Kyaik (13), Bago (14), Yangon (15), Mandalay (16), Sagaing (type locality of *C. irawadi*) (17), Sagaing (18), and Magway (19); China: Western Yunnan (20); Vietnam: Lang Son (21); China: Nanning, Guangxi (22), Hong Kong (23); China: Wanling, Hainan (24), Jiachai, Hainan (25), Tunchang, Hainan (26). Details of all samples used in this study are given in Table 1.

Seventy-three voucher specimens of *C. thailandensis* sp. nov. and *C. maehongsonensis* sp. nov. from the herpetological collection of the Chulalongkorn University Museum of Natural History (CUMZ-R) were examined for morphological analyses, and five of them were used for molecular analyses (Table 1). Additionally, seven samples of *C. thailandensis* sp. nov. and *C. maehongsonensis* sp. nov. were captured from field surveys conducted across Thailand from 2016–2019. Tissue samples were collected from tail tips for molecular analyses (Table 1). Sampling localities were selected from all geographic regions throughout Thailand, such as high mountain ranges in northwestern and western Thailand, the central plain in central Thailand, the upland plateaus in northeastern Thailand, short mountain ranges alternating with small basins in eastern Thailand, and the peninsula in southern Thailand (Fig. 1).

**Table 1.** Specimens of *Calotes* species and *Acanthosaura armata* used for morphological (A) and molecular (B) analyses and their GenBank accession number.

No.	Species	Locality	Sex	Voucher number	ND2	COI	Type of analysis	References
1	*C. thailandensis* sp. nov.	Songkhla, TH	M	CUMZ-R-2621	–	–	A	This study
2	*C. thailandensis* sp. nov.	Songkhla, TH	M	CUMZ-R-2624 (paratype)	–	–	A	This study
3	*C. thailandensis* sp. nov.	Songkhla, TH	M	CUMZ-R-2738 (holotype)	PQ212620	PQ218532	A, B	This study
4	*C. thailandensis* sp. nov.	Songkhla, TH	M	CUMZ-R-2739	–	–	A	This study
5	*C. thailandensis* sp. nov.	Songkhla, TH	M	CUMZ-R-2743	–	–	A	This study
6	*C. thailandensis* sp. nov.	Songkhla, TH	M	CUMZ-R-2769 (paratype)	–	–	A	This study
7	*C. thailandensis* sp. nov.	Songkhla, TH	M	CUMZ-R-2871 (paratype)	–	–	A	This study
8	*C. thailandensis* sp. nov.	Songkhla, TH	M	CUMZ-R-2872 (paratype)	–	–	A	This study
9	*C. thailandensis* sp. nov.	Songkhla, TH	M	CUMZ-R-2874	–	–	A	This study
10	*C. thailandensis* sp. nov.	Songkhla, TH	M	CUMZ-R-2875 (paratype)	–	–	A	This study
11	*C. thailandensis* sp. nov.	Songkhla, TH	F	CUMZ-R-2622	–	–	A	This study
12	*C. thailandensis* sp. nov.	Songkhla, TH	F	CUMZ-R-2740	–	–	A	This study
13	*C. thailandensis* sp. nov.	Songkhla, TH	F	CUMZ-R-2741	–	–	A	This study
14	*C. thailandensis* sp. nov.	Songkhla, TH	F	CUMZ-R-2742	–	–	A	This study
15	*C. thailandensis* sp. nov.	Songkhla, TH	F	CUMZ-R-2767 (paratype)	–	–	A	This study
16	*C. thailandensis* sp. nov.	Songkhla, TH	F	CUMZ-R-2768 (paratype)	–	–	A	This study
17	*C. thailandensis* sp. nov.	Songkhla, TH	F	CUMZ-R-2846 (paratype)	–	–	A	This study
18	*C. thailandensis* sp. nov.	Songkhla, TH	F	CUMZ-R-2847 (paratype)	–	–	A	This study
19	*C. thailandensis* sp. nov.	Songkhla, TH	F	CUMZ-R-2848 (paratype)	–	–	A	This study
20	*C. thailandensis* sp. nov.	Songkhla, TH	F	CUMZ-R-2850	–	–	A	This study
21	*C. thailandensis* sp. nov.	Krabi, TH	M	CUMZ-R-2703	–	–	A	This study
22	*C. thailandensis* sp. nov.	Krabi, TH	M	CUMZ-R-2708	–	–	A	This study
23	*C. thailandensis* sp. nov.	Krabi, TH	M	CUMZ-R-2711	PQ212621	PQ218533	A, B	This study
24	*C. thailandensis* sp. nov.	Krabi, TH	M	CUMZ-R-2713	–	–	A	This study
25	*C. thailandensis* sp. nov.	Krabi, TH	M	CUMZ-R-2715	–	–	A	This study
26	*C. thailandensis* sp. nov.	Krabi, TH	M	CUMZ-R-2717	–	–	A	This study
27	*C. thailandensis* sp. nov.	Krabi, TH	M	CUMZ-R-2720	–	–	A	This study
28	*C. thailandensis* sp. nov.	Krabi, TH	F	CUMZ-R-2710	–	–	A	This study
29	*C. thailandensis* sp. nov.	Krabi, TH	F	CUMZ-R-2714	–	–	A	This study
30	*C. thailandensis* sp. nov.	Krabi, TH	F	CUMZ-R-2770	–	–	A	This study
31	*C. thailandensis* sp. nov.	Krabi, TH	F	CUMZ-R-2802	–	–	A	This study
32	*C. thailandensis* sp. nov.	Krabi, TH	F	CUMZ-R-2803	–	–	A	This study
33	*C. thailandensis* sp. nov.	Krabi, TH	F	CUMZ-R-2804	–	–	A	This study
34	*C. thailandensis* sp. nov.	Krabi, TH	F	CUMZ-R-2805	–	–	A	This study
35	*C. thailandensis* sp. nov.	Krabi, TH	F	CUMZ-R-2807	–	–	A	This study
36	*C. thailandensis* sp. nov.	Krabi, TH	F	CUMZ-R-2808	–	–	A	This study
37	*C. thailandensis* sp. nov.	Ranong, TH	M	CUMZ-R-2746	–	–	A	This study
38	*C. thailandensis* sp. nov.	Ranong, TH	M	CUMZ-R-2747	–	–	A	This study
39	*C. thailandensis* sp. nov.	Ranong, TH	M	CUMZ-R-2748	–	–	A	This study
40	*C. thailandensis* sp. nov.	Ranong, TH	M	CUMZ-R-2753	–	–	A	This study
41	*C. thailandensis* sp. nov.	Ranong, TH	M	CUMZ-R-2756	–	–	A	This study
42	*C. thailandensis* sp. nov.	Ranong, TH	M	CUMZ-R-2760	–	–	A	This study
43	*C. thailandensis* sp. nov.	Ranong, TH	M	CUMZ-R-2761	–	–	A	This study
44	*C. thailandensis* sp. nov.	Ranong, TH	M	CUMZ-R-2762	–	–	A	This study
45	*C. thailandensis* sp. nov.	Ranong, TH	F	CUMZ-R-2751	–	–	A	This study
46	*C. thailandensis* sp. nov.	Ranong, TH	F	CUMZ-R-2752	–	–	A	This study
47	*C. thailandensis* sp. nov.	Ranong, TH	F	CUMZ-R-2754	–	–	A	This study
48	*C. thailandensis* sp. nov.	Ranong, TH	F	CUMZ-R-2755	–	–	A	This study
49	*C. thailandensis* sp. nov.	Ranong, TH	F	CUMZ-R-2757	–	–	A	This study
50	*C. thailandensis* sp. nov.	Ranong, TH	F	CUMZ-R-2758	–	–	A	This study
51	*C. thailandensis* sp. nov.	Ranong, TH	F	CUMZ-R-2763	–	–	A	This study
52	*C. thailandensis* sp. nov.	Ranong, TH	F	CUMZ-R-2764	–	–	A	This study
53	*C. thailandensis* sp. nov.	Chon Buri, TH	M	CUMZ-R-2905	PQ212623	PQ218535	B	This study
54	*C. thailandensis* sp. nov.	Saraburi, TH	M	CUMZ-R-2898	–	–	A	This study
55	*C. thailandensis* sp. nov.	Saraburi, TH	M	CUMZ-R-2899	PQ212622	PQ218534	B	This study
56	*C. thailandensis* sp. nov.	Kamphaeng Phet, TH	M	CUMZ-R-2933	PQ212624	PQ218536	B	This study
57	*C. thailandensis* sp. nov.	Sakon Nakhon, TH	M	CUMZ-R-2935	PQ212625	PQ218537	B	This study
58	*C. thailandensis* sp. nov.	Mukdahan, TH	M	CUMZ-R-2937	PQ212626	PQ218538	B	This study
59	*C. thailandensis* sp. nov.	Roi Et, TH	M	CUMZ-R-2939	PQ212627	PQ218539	B	This study
60	*C. maehongsonensis* sp. nov.	Mae Hong Son, TH	M	CUMZ-R-2820 (paratype)	–	–	A	This study
61	*C. maehongsonensis* sp. nov.	Mae Hong Son, TH	M	CUMZ-R-2821	–	–	A	This study
62	*C. maehongsonensis* sp. nov.	Mae Hong Son, TH	M	CUMZ-R-2822	–	–	A	This study
63	*C. maehongsonensis* sp. nov.	Mae Hong Son, TH	M	CUMZ-R-2823 (paratype)	–	–	A	This study
64	*C. maehongsonensis* sp. nov.	Mae Hong Son, TH	M	CUMZ-R-2825 (holotype)	PQ212629	PQ218541	A, B	This study
65	*C. maehongsonensis* sp. nov.	Mae Hong Son, TH	M	CUMZ-R-2826	–	–	A	This study
66	*C. maehongsonensis* sp. nov.	Mae Hong Son, TH	M	CUMZ-R-2827 (paratype)	–	–	A	This study
67	*C. maehongsonensis* sp. nov.	Mae Hong Son, TH	M	CUMZ-R-2828 (paratype)	–	–	A	This study
68	*C. maehongsonensis* sp. nov.	Mae Hong Son, TH	M	CUMZ-R-2830	–	–	A	This study
69	*C. maehongsonensis* sp. nov.	Mae Hong Son, TH	M	CUMZ-R-2832 (paratype)	–	–	A	This study
70	*C. maehongsonensis* sp. nov.	Mae Hong Son, TH	F	CUMZ-R-2824	–	–	A	This study
71	*C. maehongsonensis* sp. nov.	Mae Hong Son, TH	F	CUMZ-R-2829	–	–	A	This study
72	*C. maehongsonensis* sp. nov.	Mae Hong Son, TH	F	CUMZ-R-2831	–	–	A	This study
73	*C. maehongsonensis* sp. nov.	Mae Hong Son, TH	F	CUMZ-R-2833 (paratype)	PQ212630	PQ218542	A, B	This study
74	*C. maehongsonensis* sp. nov.	Mae Hong Son, TH	F	CUMZ-R-2834	–	–	A	This study
75	*C. maehongsonensis* sp. nov.	Mae Hong Son, TH	F	CUMZ-R-2835 (paratype)	–	–	A	This study
76	*C. maehongsonensis* sp. nov.	Mae Hong Son, TH	F	CUMZ-R-2836 (paratype)	–	–	A	This study
77	*C. maehongsonensis* sp. nov.	Mae Hong Son, TH	F	CUMZ-R-2837 (paratype)	–	–	A	This study
78	*C. maehongsonensis* sp. nov.	Mae Hong Son, TH	F	CUMZ-R-2838	–	–	A	This study
79	*C. maehongsonensis* sp. nov.	Mae Hong Son, TH	F	CUMZ-R-2839 (paratype)	PQ212631	PQ218543	A, B	This study
80	*C. maehongsonensis* sp. nov.	Kanchanaburi, TH	M	CUMZ-R-2971	PQ212628	PQ218540	B	This study
81	*C. irawadi*	Magway, MM	M	USNM562998	–	–	A	Zug et al. (2006)
82	*C. irawadi*	Magway, MM	M	CAS 213663	–	–	A	Zug et al. (2006)
83	*C. irawadi*	Magway, MM	M	CAS 213685	–	–	A	Zug et al. (2006)
84	*C. irawadi*	Magway, MM	M	CAS 213726	–	–	A	Zug et al. (2006)
85	*C. irawadi*	Magway, MM	M	CAS 213727	–	–	A	Zug et al. (2006)
86	*C. irawadi*	Magway, MM	M	CAS 213865	–	–	A	Zug et al. (2006)
87	*C. irawadi*	Magway, MM	M	CAS 213899	–	–	A	Zug et al. (2006)
88	*C. irawadi*	Magway, MM	M	CAS 216136	–	–	A	Zug et al. (2006)
89	*C. irawadi*	Sagaing, MM	M	USNM562987	–	–	A	Zug et al. (2006)
90	*C. irawadi*	Sagaing, MM	M	USNM562989	–	–	A	Zug et al. (2006)
91	*C. irawadi*	Sagaing, MM	M	USNM562990	–	–	A	Zug et al. (2006)
92	*C. irawadi*	Sagaing, MM	M	CAS 215709	–	–	A	Zug et al. (2006)
93	*C. irawadi*	Sagaing, MM	M	CAS 215423	–	–	A	Zug et al. (2006)
94	*C. irawadi*	Sagaing, MM	M	USNM520543 (holotype)	–	–	A	Zug et al. (2006)
95	*C. irawadi*	Sagaing, MM	M	CAS 215426	–	–	A	Zug et al. (2006)
96	*C. irawadi*	Sagaing, MM	M	CAS 215427	–	–	A	Zug et al. (2006)
97	*C. irawadi*	Sagaing, MM	M	CAS 215428	–	–	A	Zug et al. (2006)
98	*C. irawadi*	Sagaing, MM	M	CAS 215429	–	–	A	Zug et al. (2006)
99	*C. irawadi*	Sagaing, MM	M	CAS 215535	–	–	A	Zug et al. (2006)
100	*C. irawadi*	Sagaing, MM	M	CAS 215641	–	–	A	Zug et al. (2006)
101	*C. irawadi*	Sagaing, MM	M	CAS 215787	–	–	A	Zug et al. (2006)
102	*C. irawadi*	Sagaing, MM	M	USNM562991	–	–	A	Zug et al. (2006)
103	*C. irawadi*	Sagaing, MM	M	USNM562992	–	–	A	Zug et al. (2006)
104	*C. irawadi*	Mandalay, MM	M	USNM562996	–	–	A	Zug et al. (2006)
105	*C. irawadi*	Mandalay, MM	M	CAS 214015	–	–	A	Zug et al. (2006)
106	*C. irawadi*	Mandalay, MM	M	CAS 213954	–	–	A	Zug et al. (2006)
107	*C. irawadi*	Mandalay, MM	M	CAS 214086	–	–	A	Zug et al. (2006)
108	*C. irawadi*	Mandalay, MM	M	CAS 210605	–	–	A	Zug et al. (2006)
109	*C. irawadi*	Sagaing, MM	F	USNM520546	–	–	A	Zug et al. (2006)
110	*C. irawadi*	Sagaing, MM	F	USNM524043	–	–	A	Zug et al. (2006)
111	*C. irawadi*	Sagaing, MM	F	CAS 213891	–	–	A	Zug et al. (2006)
112	*C. irawadi*	Sagaing, MM	F	USNM562988	–	–	A	Zug et al. (2006)
113	*C. irawadi*	Sagaing, MM	F	CAS 214009	–	–	A	Zug et al. (2006)
114	*C. irawadi*	Sagaing, MM	F	CAS 231230	–	–	A	Zug et al. (2006)
115	*C. irawadi*	Sagaing, MM	F	CAS 214140	–	–	A	Zug et al. (2006)
116	*C. irawadi*	Sagaing, MM	F	USNM562993	–	–	A	Zug et al. (2006)
117	*C. irawadi*	Mandalay, MM	F	USNM563003	–	–	A	Zug et al. (2006)
118	*C. irawadi*	Mandalay, MM	F	USNM563004	–	–	A	Zug et al. (2006)
119	*C. irawadi*	Mandalay, MM	F	USNM563005	DQ289467	–	A, B	Zug et al. (2006)
120	*C. irawadi*	Mandalay, MM	F	USNM563007	–	–	A	Zug et al. (2006)
121	*C. irawadi*	Mandalay, MM	F	USNM563008	–	–	A	Zug et al. (2006)
122	*C. irawadi*	Mandalay, MM	F	CAS 210565	–	–	A	Zug et al. (2006)
123	*C. maehongsonensis* sp. nov.	Bago, MM	M	USNM563012	DQ289471	–	B	Zug et al. (2006)
124	*C. maehongsonensis* sp. nov.	Mon/Mawlamyine, MM	–	CAS 222606	DQ289472	–	B	Zug et al. (2006)
125	*C. maehongsonensis* sp. nov.	Mon/Kyaik, MM	–	USNM/fs	DQ289473	–	B	Zug et al. (2006)
126	*C. maehongsonensis* sp. nov.	Yangon, MM	–	CAS 208157	DQ289478	–	B	Zug et al. (2006)
127	*C. irawadi*	Yunnan, CN	M	NB20180905	MW591517	–	B	Liu et al. (2021)
128	*C. wangi wangi*	Lang Son, VN	–	HC201006282	KC875765	KC875765	B	Huang et al. (2023)
129	*C. wangi wangi*	Nanning, Guangxi, CN	–	hy279	KC875761	KC875761	B	Huang et al. (2023)
130	*C. wangi wangi*	Hong Kong	–	hy295	KC875772	KC875772	B	Huang et al. (2023)
131	*C. w. hainanensis*	Wanling, Hainan, CN	–	HCL200907052	KC875619	KC875619	B	Huang et al. (2023)
132	*C. w. hainanensis*	Jiachai, Hainan, CN	–	HCL200907054	KC875621	KC875621	B	Huang et al. (2023)
133	*C. w. hainanensis*	Tunchang, Hainan, CN	–	HCL200907007	KC875613	KC875613	B	Huang et al. (2023)
134	*C. versicolor*	Puducherry, IN	–	NCBS AT102	–	MZ489209	B	Gowande et al. (2021)
135	*C. versicolor*	Thiruvananthapuram, Kerala, IN	–	CESL1086	–	MZ489208	B	Gowande et al. (2021)
136	*C. emma*	Guangxi, CN	–	201607241	MZ359215	MZ359215	B	Ma et al. (2022)
137	*A. armata*	NA	–	NA	AB266452	AB266452	B	Okajima and Kumazawa (2010)

In addition, the data obtained from *Calotes irawadi* voucher specimens from Myanmar deposited at United States National Museum (**USNM**) and California Academy of Sciences (**CAS**) were provided by Dr. George R. Zug (Table 1). Sequences of *C. irawadi*, *C. wangi*, *C. versicolor*, and *C. emma* as well as of *Acanthosaura armata* were obtained from GenBank (Zug et al. 2006; Okajima and Kumazawa 2010; Gowande et al. 2021; Liu et al. 2021; Ma et al. 2022; Huang et al. 2023). All specimen data are presented in Table 1.

Tissue samples of *Calotes thailandensis* sp. nov. (*n* = 8) and *C. maehongsonensis* sp. nov. (*n* = 4) were fixed, preserved in 95% ethanol, and then stored at – 20 °C for molecular analyses (García-Muñoz et al. 2011). Total genomic DNA was extracted using BiofactTM Genomic DNA Prep Kit for Animal Tissue (BIOFACT Co., Ltd, Republic of Korea), following the protocols from the manufacturer. The target fragments, ~1000 bp of mitochondrial ND2 gene and ~1300 bp of COI gene, were amplified by polymerase chain reactions (PCR) using the forward primer L3705 (5’–ATTAGGGTCTGCTACACAAGCAGTTGG–3’) and reverse primer H5162 (5’–GGTTGARAG TARTCATCGAGTTAAGAACGA C–3’) for ND2 and forward primer L5037 (5’–GAGTAGAC CCAGGAACCRAAGTTC–3’) and reverse primer H6448 (5’–GTATACCGGCTAATCCAAGCATGTG–3’) for COI (Huang et al. 2013). PCR conditions were modified from Zug et al. (2006) and Huang et al. (2013) as follows: initial denaturation at 94 °C for 5 min, followed by denaturation for 35 cycles at 94 °C for 35 s, annealing at 58 °C for 30 s, extension at 72 °C for 1 min, and then final extension was at 72 °C for 5 min for both genes. Purified products were then sequenced using an ABI 3730XL automated DNA Sequencer. All new sequences were deposited in GenBank (accession numbers PQ212620–PQ212631 for ND2 gene and PQ218532–PQ218543 for COI gene) (Table 1).

All DNA sequences were manually checked and aligned using MUSCLE in MEGA 11 (Tamura et al. 2021). The final dataset (48 sequences) was consisted of our new ND2 (*n* = 12) and COI (*n* = 12) sequences together with ND2 (*n* = 14) and COI (*n* = 10) sequences of *Calotes* species and *Acanthosaura armata*. The concatenated phylogenetic trees comprising ND2 (948 bp) and COI sequences (1,248 bp) were constructed using maximum likelihood (ML) and Bayesian inference (BI) methods. The ML trees were constructed using IQ-TREE web server (Nguyen et al. 2015; Trifinopoulos et al. 2016) with bootstrap 1,000 replicates using the ultrafast bootstrap (UFB) analysis (Hoang et al. 2018; Minh et al. 2013). The selection of a substitution models was chosen using the Bayesian Information Criterion (BIC) in ModelFinder (Kalyaanamoorthy et al. 2017). The analyses indicated that HKY + F + G4 for 1^st^ and 2^nd^ codon positions, and TN + F + I for 3^rd^ codon position were the best fit model evolution for ND2 gene, whereas TIM2e + I for 1^st^ codon position, F81 + F for 2^nd^ codon position, and TN + F + G4 for 3^rd^ codon position were the best fit model evolution for COI gene. The branches that obtained UFB ≥ 95 were considered strongly supported (Minh et al. 2013). Following a previous study on *C. versicolor* complex by Gowande et al. (2021), branches with UFB ≥ 90 were also considered well-supported. The BI was performed using MRBAYES v. 3.2.6 (Ronquist et al. 2012) employing the best-fit model evolution using ModelFinder in Kakusan 4 (Tanabe 2011). ModelFinder identified GTR + G for the first partition, HKY85 + G for the second partition, and GTR + G for the third partition for ND2 as well as SYM + G for the first partition, HKY85 + G for the second partition, and GTR + G for the third partition for COI, respectively. The phylogenetic trees were searched using by Markov Chain Monte Carlo (MCMC) method for 10,000,000 generations, and Markov chains sampled every 1,000 generations, with 25% discarded as burn-in. When a branch or clade’s Bayesian posterior probabilities (BPP) were ≥ 0.95, it was considered to have strong, or well support (Huelsenbeck and Rannala 2004). The trees were visualized in FIGTREE v. 1.4.4 (Rambaut 2018) and edited in MEGA 11 (Tamura et al. 2021). The convergence of the runs was confirmed that the effective sample sizes (ESS) were higher than 200 by assessing the likelihood plots using TRACER v. 1.7 (Rambaut et al. 2018). Uncorrected pairwise distances (*p*-distance) based on ND2 and COI sequences were calculated within and between species of *Calotes* using MEGA 11 (Tamura et al. 2021).

Morphological analyses were performed on male (*n* = 64) and female (*n* = 51) specimens consisted of *Calotes irawadi* from central Myanmar (28 males; 14 females), *C. thailandensis* sp. nov. from central and southern Thailand (26 males; 27 females) and *C. maehongsonensis* sp. nov. from Mae Hong Son, northwestern Thailand (10 males; 10 females). Forty-one morphological characters comprising of 27 mensural characters, 11 meristic characters, and three non-meristic characters (spine length), were measured following Zug et al. (2006), Pal et al. (2018) and Prakobkarn et al. (2016).

Mensural characters were snout-vent length **(SVL)**, measured from the tip of snout to the vent; eye-ear length **(EyeEar)**, distance from anterior edge of tympanum to posterior of orbit; interorbital width **(InterOrb)**, taken from anterodorsal corners of the left to the right orbits; head height **(HeadH)**, distance from top of head to underside of jaw; head width **(HeadW)**, distance from left to right outer edge of jaw muscles at their widest point; jaw width **(JawW)**, distance from left to right outer edge of jaw angles; snout width **(SnW)**, distance between left and right nares; snout-naris length **(SnN)**, distance between tip of snout (rostral scale) and nares; snout-eye length **(SnEye)**, distance from anterior edge of orbit to tip of snout; naris-eye length **(NarEye)**, distance from anterior edge of orbit to posterior edge of naris; head length **(HeadL)**, distance from anterior edge of tympanum to tip of snout; tympanum diameter **(TymD)**, maximum diameter of the tympanum; snout-forelimb length **(SnForeL)**, distance from anterior of forelimb, or shoulder, to tip of snout; 4^th^ finger length **(4FingLng)**, distance from juncture of 3^rd^ and 4^th^ digits to tip of 4^th^ finger (excluded claw); forefoot length **(ForefL)**, distance from proximal end of forefoot to tip of fourth digit; lower arm length **(LoArmL)**, distance from elbow to distal end of wrist, or just before underside of forefoot; upper arm length **(UpArmL)**, distance from anterior insertion of forelimb, or shoulder, to elbow; 4^th^ toe length **(4ToeLng)**, distance from juncture of 3^rd^ and 4^th^ digits to tip of 4^th^ toe (excluded claw); hindfoot length **(HindfL)**, distance from proximal end (heel) of hindfoot to distalmost surface of 4^th^ toe; crus length **(CrusL)**, length of crus (tibia) from knee to heel; upper leg length **(UpLegL)**, distance from anterior edge of hindlimb insertion to knee; trunk length **(TrunkL)**, distance between posterior edge of forelimb insertion (axilla) to anterior edge of hindlimb insertion (inguen), pectoral width **(PectW)**, distance between left and right axilla (posterior to forelimb insertions) measured on ventral side; pelvic width **(PelvW)**, distance between left and right inguen (posterior to hindlimb insertions); tail height **(TailH)**, distance from dorsal to ventral surface of tail base measured just posterior to vent; the tail width **(TailW)**, distance from left to right side of tail base just posterior to vent; and the tail length **(TailL)**, distance from tail base to the tail tip.

Meristic characters were evaluated the canthus rostralis, number of elongate scales along ‘dorsolateral snout ridge’ from above posterodorsal corner of nasal scale to and including posteriormost supraciliary scale **(CanthR)**, dorsal eyelid scales **(Eyelid)**, number of scales found along dorsal edge of eyelid; dorsal head scales **(HeadSLn)**, number of scales longitudinally on midline between interparietal and rostral scale; head scales **(HeadSTr)**, number of scales in transverse line between posteriormost left and right supraciliary scales, just anterior of interparietal; infralabials **(Inflab)**, posterior end defined by posteriormost enlarged scales that touches with Suplab at rear corner of mouth; supralabials **(Suplab)**, posterior end defined by posteriormost enlarged scales that touches Inflab at rear corner of mouth; snout scales **(SnS)**, number of scales on line transversally between left and right nasal scales (single scale surrounding naris); forefoot lamellae **(4FingLm)**, number of 4^th^ finger lamellae; hindfoot lamellae **(4ToeLm)**, number of 4^th^ toe lamellae; Midbody scale rows **(Midbody)**, number of scale rows around trunk at midbody; dorsal scales **(Dorsal)**, number of middorsal scales (spines or not), beginning with first enlarged spine-like scale on nape to above vent.

Other non-meristic characters were middorsal crest spine length (**MCS)**, the length of longest middorsal (nuchal) crest spine measured from the spine based to the spine tip; anterior supratympanic spine length **(ASS)**, the length of anterior supratympanic spine measured from the spine based to the spine tip; and posterior supratympanic spine length **(PSS)**, the length of posterior supratympanic spine measured from the spine based to the spine tip. Moreover, the coloration and stripe pattern in life and preservation of the specimens were also recorded.

All characters were measured on the right side of the body. Mensural characters were taken to the closest 0.01 mm using a dial caliper, and meristic characters were counted and observed using a Zeiss Stemi DV4 stereo microscope. Sex maturity of all voucher specimens was determined from dissection: the presence of enlarge hemipenes, testis, or vas deferens in males, and presence of oviducts, or ovarian follicles in females (Meesook et al. 2016).

To examine osteological feature, two adult males of *Calotes thailandensis* sp. nov. (CUMZ-R-2738 and CUMZ-R-2769) from southern Thailand and two adult males of *C. maehongsonensis* sp. nov. (CUMZ-R-2820 and CUMZ-R-2825) from northwestern Thailand were used for this study. To obtain digital reconstruction of skulls and body for investigation, we performed high resolution X-ray micro computed tomography (CT) scans on head and body of those specimens, at Scientific and Technological Research Equipment Center of Chulalongkorn University using a SkyScan 1173® x-ray microtomography under the following settings: camera pixel size = 50 µm, voxel resolution = 7 µm, source voltage = 80 kV, source current = 100 µA. The image stacks were reconstructed using “NRecon” v. 1.6.6.0 (Bruker microCT, Kontich, Belgium). 3D surface reconstruction of skulls and pectoral girdles were constructed using 3D slicer software v. 5.0.3 (Fedorov et al. 2012) and then shapes were initially described and compared qualitatively.

All statistical analyses were performed using the statistical software in RStudio v. 1.3.1073 (RStudio Team 2020). Prior to morphological analyses, mensural characters were transformed to SVL using the following allometric equation: X_adj_ = log(X) – β[log(SVL) – log(SVL_mean_)], where X_adj_ = adjusted (size corrected) variable; X = unadjusted (measured) value; β = unstandardized regression coefficient (slope of the relationship between X and SVL) for each OTU; SVL = measured snout-vent length; SVL_mean_ = overall average SVL of all OTUs (Thorpe 1975, 1983; Turan 1999; Lleonart et al. 2000; Grismer et al. 2021). This adjustment is implemented through the allom () function in the R package GroupStruct (Chan and Grismer 2021).

In multivariate analysis, Principal Component Analysis (PCA) and Discriminant Analysis of Principal Components (DAPC) on total characters using the ADEGENET package in R (Jombart et al. 2010) were used to examine in morphological variation and the degree to which their variation coincided with species boundaries delimited by the molecular phylogenetic. Raw data of meristic characters were used for PCA and DAPC analysis. Additionally, mean differences among groups of total characters were implemented using a non-parametric permutational-multivariate analysis of variance (PERMANOVA) from *vegan* package in R.

In univariate analysis, the mean differences among groups in each character were performed and analyzed using ANOVA (for parametric data) and subsequent TukeyHSD tests with the agricolae package in R (de Mendiburu 2019) and using Kruskal–Wallis tests (for non-parametric data) with the ggsignif and ggpubr package in R (Ahlmann-Eltze and Patil 2021). Boxplots of raw morphological data were generated using the ggplot2 package in R to visualize the range and degree of differences of characters bearing statistical differences between groups.

### Species delimitation

Species delimitation of *Calotes thailandensis* sp. nov. and *C. maehongsonensis* sp. nov. in Thailand follows the strategy proposed by de Queiroz (1998, 2007), Vijayakumar et al. (2014), Shanker et al. (2017), Burbrink and Ruane (2021). In the initial step, species delimitation of this species complex group was performed on both ND2 and COI genes following Zug et al. (2006) and Gowande et al. (2021) and the trees were constructed using ML and BI methods with outgroup dropped (such as described *C. versicolor* from type locality in India). Monophyletic group is acceptable with strong bootstrap value (≥ 90) (Gowande et al. 2021) and posterior probability value (≥ 0.90) (Gowande et al. 2021), coupled with species-level divergences within *Calotes*, for example, genetic distance is as high as, or excessed genetic distance between *C. thailandensis* sp. nov. and *C. maehongsonensis* sp. nov. compared to *C. irawadi* and *C. wangi* (~ 4%) (Zug et al. 2006; Huang et al. 2023). Then, diagnostic accounts of differentiation species are also considered based on morphological characters. Finally, we also consider the allopatric distribution of those groups as new species because they are in complete reproductive isolation, and then allopatric speciation occurred.

## Results

### Phylogenetic relationships

**Figure 2.**
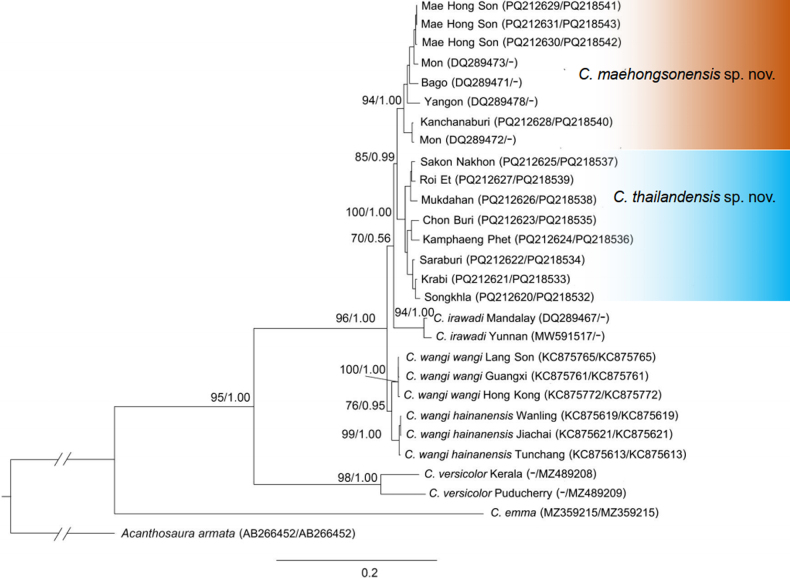
The maximum likelihood (ML) tree of *Calotes* species (*n* = 27) and *Acanthosaura armata* was used as an outgroup based on the mitochondrial NADH dehydrogenase subunit 2 (ND2; 948 bps) and the mitochondrial cytochrome c oxidase subunit I (COI; 1,248 bps) genes with ultrafast bootstrap (UFB)/Bayesian posterior probabilities (BPP) supports. GenBank accession numbers of the ND2 and COI genes are shown in the bracket, respectively (see Table 1).

To assess the phylogenetic relationship among the two proposed new species, *Calotes thailandensis* sp. nov. (*n* = 8) and *C. maehongsonensis* sp. nov. (*n* = 8), and other *Calotes* species (*n* = 11), the mitochondrial ND2 (948 bps, 50 parsimony informative sites) and COI (1,245 bps, 76 parsimony informative sites) genes were analyzed. Maximum likelihood (ML) and Bayesian inference (BI) phylogenetic trees were constructed from concatenated ND2 and COI sequences (2,193 bps) (Fig. 2, Table 1), the results of which reveal similar topologies, where *Calotes* samples from Thailand, Myanmar and China are grouped together within a major clade, clearly separated from Indian *C. versicolor*. Most importantly, the phylogenetic tree reveals distinct groupings consisting of clades of *C. irawadi* and *C. wangi* clades, separated from *C. thailandensis* sp. nov. clade and *C. maehongsonensis* sp. nov. clade. Both clades containing *C. thailandensis* sp. nov. from central, southern, and northeastern Thailand (100% for MLUFB; 1.00 for BPP) and *C. maehongsonensis* sp. nov. from northwestern Thailand and southeastern Myanmar including Mon, Bago, and Yangon (94% for MLUFB; 1.00 for BPP) are strongly supported (Fig. 2).

In addition to the distinct groupings of the four minor clades, the analyses of uncorrected pairwise genetic distances (*p*-distances) among samples reveal values ranging from 3.34–7.08% for ND2 gene (Table 2) and from 3.72–5.36% for COI gene (Table 3). Evidently, the ND2 genetic distances between the clades containing *C. thailandensis* sp. nov. and *C. maehongsonensis* sp. nov., and the true *C. irawadi* clade range from 4.94–7.08%, which are slightly greater than the interspecific genetic distances between *C. wangi* and two new species from Thailand (4.03–5.86% for ND2 gene and 3.72–5.36% for COI gene).

**Table 2.** Uncorrected *p*-distances (%) based on ND2 mtDNA sequences (948 bps) between and within species of *Calotes*.

Species	*n*	*C. thailandensis* sp. nov.	*C. maehongsonensis* sp. nov.	*C. wangi wangi*	*C. wangi hainanensis*	*C. irawadi*	*C. emma* (MZ359215)
*C. thailandensis* sp. nov.	8	2.11					
		(0.63–2.90)					
*C. maehongsonensis* sp. nov.	8	4.06	1.94				
		(3.34–5.05)	(0.00–3.02)				
*C. wangi wangi*	3	4.62	4.55	0.07			
		(4.26–5.03)	(4.03–5.05)	(0.00–0.11)			
*C. wangi hainanensis*	3	4.91	5.01	1.78	0.21		
		(4.35–5.27)	(4.48–5.86)	(1.71–1.93)	(0.11–0.32)		
*C. irawadi*	2	5.69	5.94	6.36	6.07	1.27	
		(4.94–6.38)	(5.55–7.08)	(5.98–6.79)	(5.64–6.52)		
*C. emma* (MZ359215)	1	38.49	38.84	38.85	38.76	36.32	NA
		(37.91–39.24)	(38.30–39.39)	(38.78–38.98)	(38.59–38.88)	(33.82–38.83)	

**Table 3.** Uncorrected *p*-distances (%) based on COI mtDNA sequences (1,248 bps) between and within species of *Calotes*.

Species	*n*	*C. thailandensis* sp. nov.	*C. maehongsonensis* sp. nov.	*C. wangi wangi*	*C. wangi hainanensis*	*C. versicolor* (MZ489209)	*C. emma* (MZ359215)
*C. thailandensis* sp. nov.	8	2.29					
		(0.81–3.20)					
*C. maehongsonensis* sp. nov.	4	4.33	1.4				
		(3.97–4.83)	(0.00–2.70)				
*C. wangi wangi*	3	4.52	4.16	0.32			
		(3.72–5.09)	(3.98–4.32)	(0.24–0.40)			
*C. wangi hainanensis*	3	4.58	3.98	2.07	0.32		
		(4.15–5.36)	(3.72–4.15)	(1.96–2.21)	(0.08–0.48)		
*C. versicolor* (MZ489209)	1	15.61	16.16	16.84	16.06	NA	
		(15.31–16.16)	(15.95–16.37)	(16.55–17.19)	(15.92–16.13)		
*C. emma* (MZ359215)	1	21.08	19.95	21.29	21.54	21.61	NA
		(20.57–21.64)	(19.68–20.53)	(21.25–21.37)	(21.41–21.68)		

### Morphological analyses

On account of specimen availability and sample sizes, morphological examinations and comparisons were conducted based on two new species of *Calotes*, *C. thailandensis* sp. nov. and *C. maehongsonensis* sp. nov., from Thailand and previously obtained data of *C. irawadi* from central Myanmar (Zug et al. 2006).

**Figure 3.**
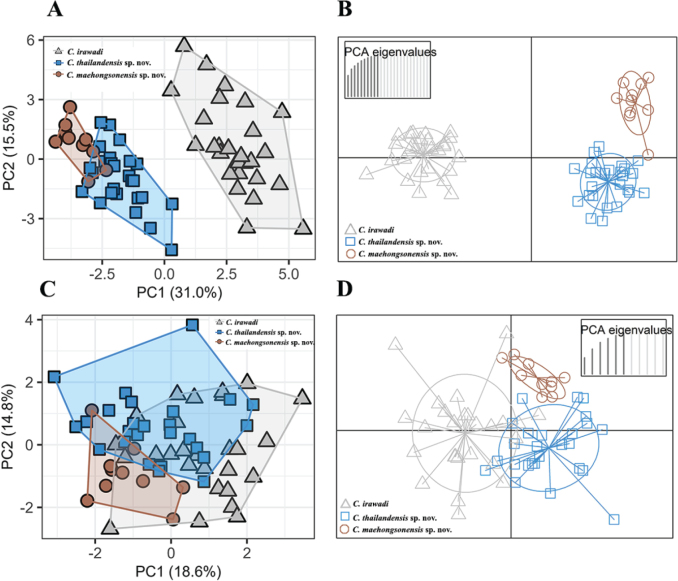
Males: PCA (**A, C**) and DAPC (**B, D**) of male *Calotes* species based on mensural (**A, B**) and meristic (**C, D**) characters, respectively, containing *C. irawadi* from central Myanmar (*n* = 28), *C. thailandensis* sp. nov. from central and southern Thailand (*n* = 26) and *C. maehongsonensis* sp. nov. from Mae Hong Son, northwestern Thailand (*n* = 10). PERMANOVA support the significantly morphological difference with *p* < 0.001.

In males, both multivariate analyses, PCA and DAPC, demonstrate distinct morphological differences in mensural and meristic characters among *C. irawadi* from central Myanmar and two new species from Thailand containing *C. maehongsonensis* sp. nov. from northwestern (Mae Hong Son) Thailand and *C. thailandensis* sp. nov. from central and southern Thailand (Fig. 3). Although the results are based on meristic characters (Fig. 3C, D), the particular PCA reveals marginal overlapping among three *Calotes* species, analyses of mensural characters reveal well-defined separation (Fig. 3A, B). The cumulative variance of the first three principal components from mensural and meristic analyses are 55.5% (31.0% for PC1, 15.5% for PC2 and 9.0% for PC3; Suppl. material 1) and 46.3% (18.6% for PC1, 14.8% for PC2 and 12.9% for PC3; Suppl. material 2), respectively. In addition, the results of PERMANOVA clearly reveal significant difference in mensural characters (*F* = 35.64; *R*^2^ = 0.54; *p* < 0.001) as well as meristic characters (*F* = 8.16; *R*^2^ = 0.21; *p* < 0.001).

Regarding univariate analyses, although most characters broadly overlap, 20 of 25 adjusted mensural characters and five of the 11 meristic characters have significantly different mean values when comparing specimens from the three representative clades (Suppl. material 5: figs S1, S2, Tables 4, 5), particularly HeadH, JawW, SnN, SnForeL, TrunkL, PelvW, TailH, and TailW. Head size measurements (HeadH, JawW, SnN, and SnForeL) are largest in *C. irawadi* and smallest in *C. maehongsonensis* sp. nov., whereas tail measurements (TailH, and TailW) are largest in *C. maehongsonensis* sp. nov. and smallest in *C. irawadi*. Noticeably, males *C. thailandensis* sp. nov. and *C. maehongsonensis* sp. nov. from Thailand are distinguished from male *C. irawadi* from central Myanmar by distinct larger PelvW and higher numbers of HeadSLn. *Calotes maehongsonensis* sp. nov. is distinguished from others by smaller HeadH, SnN, SnForeL, and TailH, whereas *C. thailandensis* sp. nov. differ significantly from others by having fewer Midbody and Dorsal.

**Table 4.** Morphological (mensural) characters of holotype, paratypes and all specimens of *Calotes thailandensis* sp. nov., *C. maehongsonensis* sp. nov., *C. irawadi* and *C. wangi*. Measurements are represented in mean ± SD (range) in millimeters (mm). Abbreviations are showed in Materials and methods.

Characters	*C. thailandensis* sp. nov.					*C. maehongsonensis* sp. nov.					*C. irawadi*		*C. wangi*	
	Holotype	Paratypes		All specimens		Holotype	Paratypes		All specimens		All specimens		Huang et al. (2023)	
	Male	Male (*n =* 5)	Female (*n =* 5)	Male (*n =* 26)	Female (*n =* 27)	Male	Male (*n =* 5)	Female (*n =* 5)	Male (*n =* 10)	Female (*n =* 10)	Male (*n =* 28)	Female (*n =* 14)	Male (*n =* 2)	Female (*n =* 2)
SVL	87.24	85.37 ± 1.22	77.08 ± 3.57	87.11 ± 4.80	74.93 ± 4.41	76.16	78.47 ± 1.28	74.51 ± 4.14	78.81 ± 2.48	73.36 ± 4.68	82.59 ± 8.32	77.40 ± 7.91		
		(83.87–86.30)	(70.87–79.53)	(80.90–99.74)	(65.30–82.91)		(76.86–79.6)	(70.71–80.80)	(76.16–83.38)	(67.36–80.80)	(66.40–106.80)	(64.30–90.30)	(88.34–96.4)	(82–96.5)
EyeEar	6.25	6.36 ± 0.61	4.46 ± 0.23	6.39 ± 0.52	4.54 ± 0.36	4.8	5.35 ± 0.42	4.42 ± 0.23	5.32 ± 0.41	4.35 ± 0.20	5.76 ± 0.89	4.30 ± 0.60		
		(5.63–7.10)	(4.20–4.80)	(5.24–7.28)	(3.98–5.53)		(4.7–5.8)	(4.19–4.74)	(4.70–5.90)	(4.14–4.74)	(4.20–7.90)	(3.10–5.50)	(5.97–6.30)	(5.06–6.17)
InterOrb	8.6	8.93 ± 0.36	7.43 ± 0.24	8.80 ± 0.49	7.33 ± 0.59	7.42	7.88 ± 0.46	6.93 ± 0.72	7.80 ± 0.39	6.89 ± 0.64	9.40 ± 0.91	8.33 ± 1.05		
		(8.48–9.35)	(7.14–7.68)	(7.81–9.70)	(6.30–8.73)		(7.14–8.4)	(6.56–8.22)	(7.14–8.40)	(6.38–8.22)	(7.90–11.30)	(6.90–10.70)	(8.51–9.48)	(10.24–12.43)
HeadH	15.25	14.99 ± 0.83	11.49 ± 0.86	14.18 ± 1.26	11.23 ± 0.80	11.66	11.78 ± 0.66	10.93 ± 0.43	11.70 ± 0.53	10.75 ± 0.41	14.82 ± 2.19	12.96 ± 1.55		
		(14.28–16.40)	(10.85–12.85)	(11.70–16.40)	(10.00–12.85)		(11.12–12.9)	(10.38–11.30)	(11.10–12.90)	(10.10–11.30)	(11.20–21.50)	(8.90–15.00)	(13.18–14.42)	(12.42–14.42)
HeadW	20.18	19.55 ± 2.61	13.71 ± 0.16	19.69 ± 1.83	13.19 ± 0.81	16.1	15.82 ± 1.89	12.70 ± 0.76	15.68 ± 1.43	12.63 ± 0.83	19.09 ± 3.67	14.31 ± 1.92		
		(16.00–21.95)	(13.50–13.90)	(16.00–23.22)	(11.70–14.70)		(13.00–18.30)	(11.80–13.90)	(13.00–18.30)	(11.55–13.90)	(13.30–28.30)	(11.20–17.80)	(15.42–16.09)	(14.21–14.79)
JawW	14.21	13.85 ± 1.05	11.91 ± 0.47	14.41 ± 1.08	11.60 ± 0.81	12.24	12.54 ± 0.26	11.43 ± 0.89	12.56 ± 0.56	11.26 ± 0.78	15.85 ± 2.76	12.58 ± 1.54		
		(12.74–15.05)	(11.30–12.38)	(12.40–16.82)	(10.20–13.46)		(12.31–12.97)	(10.70–12.80)	(11.54–13.71)	(10.60–12.80)	(11.70–22.50)	(9.50–15.20)	(16.06–16.14)	(13.47–16.84)
SnW	5.27	5.24 ± 0.35	4.63 ± 0.06	5.36 ± 0.34	4.74 ± 0.44	4.12	4.58 ± 0.23	4.59 ± 0.25	4.56 ± 0.23	4.47 ± 0.32	5.32 ± 0.51	5.18 ± 0.45		
		(4.74–5.60)	(4.54–4.68)	(4.74–6.15)	(4.04–5.70)		(4.36–4.92)	(4.36–4.95)	(4.12–4.92)	(3.91–4.95)	(4.30–6.50)	(4.40–5.90)	(5.29–5.87)	(5.33–5.76)
SnN	3.65	3.86 ± 0.20	3.47 ± 0.20	3.93 ± 0.26	3.41 ± 0.27	3	3.43 ± 0.16	3.44 ± 0.34	3.37 ± 0.19	3.36 ± 0.32	4.19 ± 0.63	3.84 ± 0.55		
		(3.62–4.10)	(3.23–3.78)	(3.53–4.54)	(2.92–4.09)		(3.23–3.6)	(3.10–3.92)	(3.00–3.60)	(3.04–3.92)	(2.80–5.40)	(2.60–4.70)		
SnEye	8.7	8.68 ± 0.42	7.57 ± 0.34	8.83 ± 0.35	7.49 ± 0.44	7.55	7.94 ± 0.46	7.45 ± 0.59	7.88 ± 0.42	7.33 ± 0.61	8.50 ± 0.87	7.81 ± 0.79		
		(8.08–9.04)	(7.14–7.93)	(8.08–9.48)	(6.73–8.50)		(7.38–8.5)	(6.66–8.20)	(7.38–8.50)	(6.43–8.20)	(6.90–10.70)	(5.90–9.10)	(7.85–8.82)	(7.91–9.0)
NarEye	4.48	4.78 ± 0.36	3.91 ± 0.19	4.85 ± 0.30	3.96 ± 0.28	4.34	4.47 ± 0.32	4.13 ± 0.32	4.39 ± 0.25	3.97 ± 0.31	4.32 ± 0.41	3.96 ± 0.37		
		(4.42–5.37)	(3.70–4.16)	(4.28–5.37)	(3.52–4.57)		(4.05–4.86)	(3.92–4.70)	(4.05–4.86)	(3.58–4.70)	(3.70–5.30)	(3.30–4.50)	(4.49–4.58)	(4.96–5.69)
HeadL	20.95	20.57 ± 1.22	17.25 ± 0.87	21.26 ± 1.18	17.16 ± 1.13	17.87	18.68 ± 0.71	16.68 ± 1.53	18.68 ± 0.75	16.15 ± 1.32	20.03 ± 1.83	17.64 ± 1.83		
		(19.16–22.18)	(16.10–18.46)	(19.50–23.52)	(15.37–19.87)		(17.88–19.36)	(14.42–18.44)	(17.87–19.65)	(14.42–18.44)	(16.90–24.90)	(13.90–21.40)	(24.29–24.47)	(23.39–23.93)
SnForeL	30.38	29.14 ± 2.23	21.74 ± 1.20	29.15 ± 1.74	21.73 ± 1.40	23.72	24.58 ± 1.14	21.57 ± 1.70	24.86 ± 1.18	21.20 ± 1.51	29.19 ± 2.24	26.55 ± 3.32		
		(25.74–31.20)	(20.44–23.60)	(25.74–32.40)	(19.46–25.08)		(23.1–25.87)	(20.03–24.06)	(23.10–26.70)	(19.40–24.06)	(25.40–33.70)	(20.80–32.10)	(30.74–32.65)	(25.99–30.4)
4FingLng	10.16	10.27 ± 0.87	8.47 ± 0.34	10.42 ± 0.66	8.36 ± 0.35	8.8	9.73 ± 0.53	8.46 ± 0.63	9.48 ± 0.49	8.19 ± 0.64	10.64 ± 1.01	9.91 ± 0.91		
		(9.00–11.17)	(7.97–8.87)	(9.00–11.36)	(7.50–8.92)		(9.15–10.46)	(7.72–9.42)	(8.80–10.46)	(7.32–9.42)	(8.70–12.80)	(8.70–11.50)	(13.41–14.10)	(9.82–13.68)
ForefL	14.92	14.41 ± 1.19	11.89 ± 0.39	14.75 ± 1.14	11.96 ± 0.76	13.15	13.19 ± 0.63	11.61 ± 0.90	13.02 ± 0.55	11.47 ± 0.64	14.95 ± 1.44	13.92 ± 1.14		
		(12.93–16.16)	(11.37–12.22)	(12.24–16.75)	(10.04–13.50)		(12.25–13.80)	(10.24–12.74)	(12.24–13.80)	(10.24–12.74)	(12.10–18.70)	(11.60–16.00)		
LoArmL	15.66	15.01 ± 0.50	13.17 ± 1.33	14.99 ± 0.74	12.32 ± 0.92	12.03	13.95 ± 0.49	12.50 ± 0.66	13.85 ± 0.75	12.36 ± 0.62	15.83 ± 1.69	13.86 ± 1.50		
		(14.54–15.80)	(11.36–15.00)	(13.62–16.80)	(10.30–15.00)		(13.21–14.36)	(11.50–13.16)	(12.03–14.38)	(11.41–13.16)	(13.10–21.10)	(10.10–16.00)	(13.59–14.34)	(13.6–15.11)
UpArmL	17.26	15.68 ± 0.86	12.82 ± 0.66	15.15 ± 1.05	12.16 ± 0.62	13.47	13.90 ± 0.58	12.29 ± 0.45	13.98 ± 0.51	12.03 ± 0.65	17.06 ± 2.03	15.19 ± 2.15		
		(14.36–16.63)	(12.26–13.88)	(13.50–17.42)	(11.00–13.88)		(13.29–14.74)	(11.72–12.88)	(13.29–14.76)	(10.90–12.88)	(13.40–21.70)	(11.80–19.90)	(15.8–16.64)	(15.35–16.36)
4ToeLng	16.4	16.27 ± 0.54	13.23 ± 0.54	16.17 ± 0.81	13.38 ± 0.54	13.8	14.29 ± 1.05	12.96 ± 0.90	14.34 ± 0.83	12.54 ± 0.82	16.27 ± 1.72	14.44 ± 0.86		
		(15.68–17.13)	(12.72–14.00)	(14.24–18.00)	(12.54–14.20)		(13.33–15.89)	(11.96–14.11)	(13.33–15.89)	(11.56–14.11)	(13.80–20.70)	(13.30–16.00)	(19.04–21.57)	(15.36–17.97)
HindfL	25.92	26.04 ± 0.65	21.63 ± 0.50	25.87 ± 1.46	21.61 ± 0.98	21.02	23.11 ± 1.47	20.98 ± 1.64	23.26 ± 1.43	20.44 ± 1.33	26.83 ± 2.29	24.52 ± 1.59		
		(25.19–26.65)	(21.09–22.36)	(22.80–28.80)	(19.76–23.64)		(21.48–25.12)	(18.80–23.18)	(21.02–25.20)	(18.80–23.18)	(23.00–34.10)	(21.00–26.50)		
CrusL	19.18	19.20 ± 0.72	16.13 ± 0.68	18.94 ± 0.88	15.75 ± 0.73	16.72	17.51 ± 0.53	15.80 ± 0.88	17.71 ± 0.86	15.50 ± 0.85	19.45 ± 1.67	17.70 ± 1.38		
		(18.56–20.30)	(15.10–16.90)	(17.33–20.85)	(14.16–17.35)		(16.74–18.21)	(14.86–17.07)	(16.72–19.53)	(14.33–17.07)	(16.50–24.80)	(14.00–19.40)	(16.87–20.26)	(17.38–18.08)
UpLegL	21.86	21.61 ± 0.81	18.39 ± 0.64	21.05 ± 1.10	17.77 ± 0.94	19.2	19.37 ± 0.38	17.44 ± 1.12	19.40 ± 0.70	17.25 ± 0.82	20.16 ± 1.95	18.12 ± 1.52		
		(20.78–22.78)	(17.36–19.00)	(18.54–22.78)	(15.98–20.00)		(19.04–19.84)	(16.07–18.73)	(18.23–20.94)	(16.07–18.73)	(16.80–25.50)	(14.50–20.90)	(21.94–23.01)	(17.27–20.76)
TrunkL	42.93	39.87 ± 0.88	37.80 ± 2.66	40.97 ± 2.97	36.08 ± 2.74	34.54	36.80 ± 1.40	35.93 ± 2.41	36.76 ± 2.14	35.59 ± 3.27	41.67 ± 5.41	40.61 ± 4.34		
		(38.75–40.79)	(33.26–40.20)	(37.27–49.10)	(30.16–40.51)		(35.48–38.78)	(33.26–38.53)	(34.24–41.42)	(31.14–39.73)	(31.70–56.90)	(32.10–44.90)	(43.82–49.82)	(40.81–47.02)
PectW	14.74	15.35 ± 1.48	12.18 ± 0.35	15.28 ± 1.48	11.81 ± 0.95	13.38	13.37 ± 0.91	11.90 ± 0.64	13.52 ± 0.94	12.05 ± 1.02	14.61 ± 2.45	12.28 ± 1.68		
		(12.90–16.72)	(11.75–12.56)	(12.90–19.76)	(10.12–13.93)		(12.45–14.38)	(11.10–12.86)	(12.44–15.33)	(11.07–14.40)	(9.80–21.40)	(9.50–14.70)		
PelvW	14.26	13.28 ± 0.67	11.31 ± 0.99	13.16 ± 0.93	11.27 ± 0.99	11.33	11.46 ± 0.91	11.61 ± 1.06	11.38 ± 0.67	11.52 ± 0.87	8.59 ± 0.98	8.15 ± 0.87		
		(12.58–14.09)	(10.15–12.54)	(11.62–15.07)	(9.51–13.44)		(10.46–12.95)	(10.20–12.88)	(10.46–12.95)	(10.20–12.88)	(6.90–10.90)	(6.80–9.30)		
TailH	13.35	12.56 ± 0.52	6.19 ± 0.33	12.41 ± 1.13	6.18 ± 0.59	10.72	11.12 ± 0.73	6.19 ± 0.43	11.48 ± 0.83	6.23 ± 0.48	10.11 ± 1.43	7.37 ± 1.48		
		(12.12–13.38)	(5.82–6.54)	(9.55–14.83)	(5.34–7.30)		(10.05–11.79)	(5.70–6.62)	(10.05–12.74)	(5.37–6.84)	(7.80–14.20)	(4.70–10.20)		
TailW	11.61	11.27 ± 0.72	9.34 ± 0.30	10.78 ± 0.85	8.73 ± 0.94	9.37	10.36 ± 0.65	8.51 ± 0.59	10.32 ± 0.68	8.72 ± 0.58	9.69 ± 1.28	6.94 ± 1.34		
		(10.62–12.32)	(9.00–9.81)	(9.26–12.92)	(7.02–10.87)		(9.30–11.02)	(7.78–9.40)	(9.30–11.40)	(7.78–9.63)	(7.30–13.40)	(5.30–10.50)		
TailL*	253	250.8 ± 4.7	224 ± 14.6	254.9 ± 11.2	212.30 ± 17.47	210	222.0 ± 15.6	188.5 ± 16.2	221.9 ± 10.5	186.8 ± 15.4	231.52 ± 20.73	211.4 ± 21.5		
		(24 5–254)	(215–250)	(236–278)	(174–250)		(211–233)	(175–212)	(210–233)	(170–212)	(191–288)	(182–250)	(256.15–283.90)	(243.5–330)

*TailL of male paratypes from *C. thailandensis* sp. nov. (*n* = 4) and *C. maehongsonensis* sp. nov. (*n* = 2); TailL of female paratypes from *C. thailandensis* sp. nov. (*n* = 5) and *C. maehongsonensis* sp. nov. (*n* = 4).

**Table 5.** Morphological (meristic) characters of holotype, paratypes and all specimens of *Calotes thailandensis* sp. nov., *C. maehongsonensis* sp. nov., *C. irawadi*, and *C. wangi*. Numbers of scales are represented in mean ± SD (range). Abbreviations are showed in Materials and methods.

Characters	*C. thailandensis* sp. nov.					*C. maehongsonensis* sp. nov.					*C. irawadi*		*C. wangi*
	Holotype	Paratypes		All specimens		Holotype	Paratypes		All specimens		All specimens		Huang et al. (2023)
	Male	Male (*n* = 5)	Female (*n* = 5)	Male (*n* = 26)	Female (*n* = 27)	Male	Male (*n* = 5)	Female (*n* = 5)	Male (*n* = 10)	Female (*n* = 10)	Male (*n* = 28)	Female (*n* = 14)	Male (*n* = 2)
CanthR	8	7.8 ± 0.4	7.6 ± 0.6	8.0 ± 0.5	7.6 ± 0.5	8	8.2 ± 0.4	8 ± 0.0	8.2 ± 0.4	8.0 ± 0	7.9 ± 0.6	7.7 ± 0.5	4.91*
		(7–8)	(7–8)	(7–9)	(7–8)		(8–9)	8	(8–9)	8	(6–9)	(7–8)	(4–5)
Eyelid	11	12.2 ± 1.3	9.8 ± 0.8	12.2 ± 1.1	9.6 ± 1.1	14	12.4 ± 0.5	9.2 ± 1.3	12.4 ± 1.1	9.5 ± 1.1	12.7 ± 1.4	11.9 ± 0.7	12.4*
		(11–14)	(9–11)	(10–14)	(8–12)		(12–13)	(8–11)	(11–14)	(8–11)	(10–15)	(11–13)	(10–14)
HeadSLn	13	13.4 ± 1.7	12.6 ± 0.6	13.4 ± 1.0	13.2 ± 1.3	14	14.4 ± 0.5	12.8 ± 1.8	14.3 ± 0.5	13.4 ± 1.6	12.1 ± 1.2	14.0 ± 1.5	
		(12–16)	(12–13)	(12–16)	(11–17)		(14–15)	(11–15)	(14–15)	(11–16)	(10–14)	(12–17)	
HeadSTr	15	13.0 ± 0.7	13.4 ± 1.5	13.8 ± 1.0	13.0 ± 1.3	13	13.0 ± 0.7	12 ± 0.7	13.1 ± 0.7	12.8 ± 2.3	13.9 ± 1.2	12.0 ± 1.0	
		(12–14)	(11–15)	(12–15)	(11–16)		(12–14)	(11–13)	(12–14)	(11–19)	(12–16)	(10–13)	
Inflab	10	10.0 ± 0.0	9.6 ± 0.9	10.1 ± 0.7	9.3 ± 0.6	10	10.4 ± 0.5	9.4 ± 0.9	10.1 ± 0.7	9.2 ± 0.9^b^	10.3 ± 0.7	10.1 ± 1.1	
		10	(9–11)	(9–11)	(8–11)		(10–11)	(8–10)	(9–11)	(8–10)	(9–12)	(9–12)	(9–12)
Suplab	10	10.4 ± 0.5	9.2 ± 0.5	10.5 ± 0.6	9.1 ± 0.5	11	11.0 ± 0.0	9.8 ± 0.8	10.8 ± 0.4	9.7 ± 0.7	10.9 ± 0.7	10.6 ± 0.9	
		(10–11)	(9–10)	(9–12)	(8–10)		11	(9–11)	(10–11)	(9–11)	(9–12)	(9–13)	(9–11)
SnS	7	6.4 ± 0.5	6.2 ± 0.5	6.76 ± 0.7	6.4 ± 0.5	5	6.0 ± 0.7	6.2 ± 0.4	6.0 ± 0.7	6.1 ± 0.3	6.8 ± 0.6	6.9 ± 0.8	
		(6–7)	(6–7)	(6–8)	(6–7)		(5–7)	(6–7)	(5–7)	(6–7)	(6–8)	(6–8)	
4FingLm	21	19.8 ± 1.1	19.6 ± 1.1	20.1 ± 1.2	19.4 ± 1.0	19	19.8 ± 0.8	19 ± 1.6	19.5 ± 0.8	18.6 ± 1.3	20.9 ± 1.4	19.4 ± 1.4	
		(19–21)	(18–21)	(18–22)	(17–21)		(19–21)	(17–21)	(18–21)	(17–21)	(18–24)	(17–22)	(20–22)
4ToeLm	23	25.2 ± 2.2	25.0 ± 1.0	25.1 ± 1.7	24.8 ± 1.3	23	24.6 ± 1.1	23.4 ± 1.5	24.2 ± 1.1	22.5 ± 1.5^b^	25.3 ± 1.6	24.1 ± 1.4	24.2*
		(23–28)	(24–26)	(23–29)	(23–28)		(23–26)	(21–25)	(23–26)	(21–25)	(23–29)	(22–28)	(19–27)
Midbody	43	41.6 ± 2.7	42.4 ± 1.8	42.5 ± 2.0	44.3 ± 3.0	44	44.6 ± 0.5	44.2 ± 2.5	44.5 ± 0.8	43.5 ± 2.0	45.4 ± 2.1	46.4 ± 2.3	41.2*
		(37–44)	(40–44)	(37–46)	(39–51)		(44–45)	(42–48)	(43–46)	(41–48)	(42–50)	(43–51)	(36–46)
Dorsal	46	46.6 ± 1.8	46.6 ± 1.7	46.0 ± 1.9	47.2 ± 1.8	51	48.2 ± 1.8	51 ± 2.4	48.8 ± 1.5	50.8 ± 2.1^a^	48.3 ± 2.4	51.6 ± 4.5	12.4
		(45–49)	(45–49)	(40–49)	(44–50)		(47–51)	(48–54)	(47–51)	(48–54)	(41–53)	(45–59)	(38–44)

*unknown sample size.

**Figure 4.**
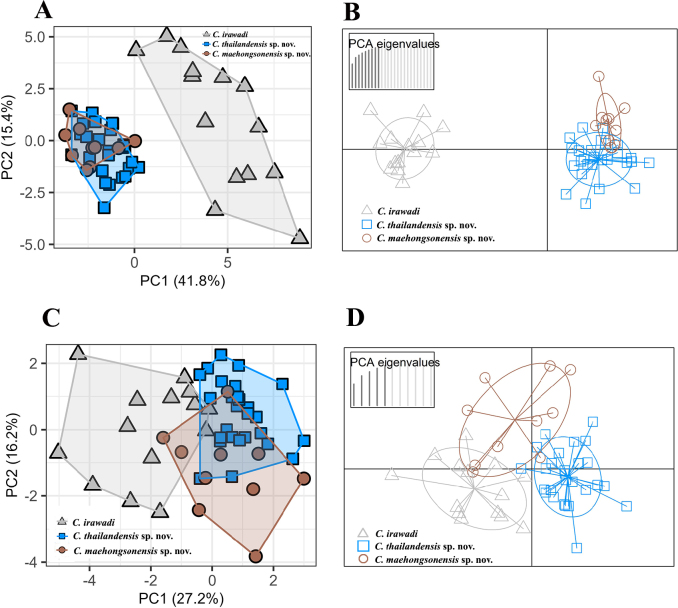
Females: PCA (**A, C**) and DAPC (**B, D**) of female *Calotes* species based on mensural (**A, B**) and meristic (**C, D**) characters, respectively, containing *C. irawadi* from central Myanmar (*n* = 14), *C. thailandensis* sp. nov. from central and southern Thailand (*n* = 27) and *C. maehongsonensis* sp. nov. from Mae Hong Son, northwestern Thailand (*n* = 10). PERMANOVA support the significantly morphological difference with *p* < 0.001.

As well as the PCA and DAPC analyses of male characters revealing distinct separation between the three species, analyses of female characters also reveal distinct separation of *C. irawadi* from the two new species (Fig. 4). The PERMANOVA results reveal significant differences in mensural characters among three *Calotes* species (*F* = 23.69; *R*^2^ = 0.50; *p* < 0.001) and significant differences in meristic characters between two new species from Thailand (*F* = 8.45; *R*^2^ = 0.26; *p* < 0.001). The cumulative variance of the first three principal components from mensural and meristic analyses are 63.6% (41.8% for PC1, 15.4% for PC2 and 6.4% for PC3; Suppl. material 1) and 56.7% (27.2% for PC1, 16.2% for PC2 and 13.3% for PC3; Suppl. material 2), respectively.

Univariate analyses of female characters reveal significant differences among 17 of 25 adjusted mensural characters (Suppl. material 5: fig. S3, Table 4) and eight of 11 meristic characters (Suppl. material 5: fig. S4, Table 5). Female specimens from the three aforementioned species are distinguished from each other by significant differences in 4ToeLng, and HindfL, where *C. maehongsonensis* sp. nov. are smallest, and *C. irawadi* from central Myanmar display the largest values. Although female *C. thailandensis* sp. nov. and *C. maehongsonensis* sp. nov. are hardly distinguishable in mensural characters, they are separated by numbers of CanthR, 4ToeLm and Dorsal, such that *C. maehongsonensis* sp. nov. having greater numbers of CanthR and Dorsal, but fewer numbers of 4ToeLm.

Further morphological examinations of spines and skeletons of *C. thailandensis* sp. nov. and *C. maehongsonensis* sp. nov. reveal substantial and significant differences in the lengths of the anterior and posterior supratympanic spines. In *C. thailandensis* sp. nov., the ratio of the anterior supratympanic spine (ASS) length to the tympanum diameter is 0.58 ± 0.13, which is significantly longer compared to *C. maehongsonensis* sp. nov., the ratio of which is 0.38 ± 0.11 (*t* = 4.47; *p* < 0.001). Likewise, the ratio of the posterior supratympanic spine (PSS) length to the tympanum diameter of *C. thailandensis* sp. nov. is 0.53 ± 0.12, which is also longer compared to *C. maehongsonensis* sp. nov., which is 0.43 ± 0.07 (*t* = 3.21; *p* = 0.003). In contrast, comparison of middorsal crest spine (MCS) length to tympanum diameter ratio reveals no significant difference between *C. thailandensis* sp. nov. and *C. maehongsonensis* sp. nov. (*t* = – 0.91; *p* = 0.374) (Suppl. material 4).

**Figure 5.**
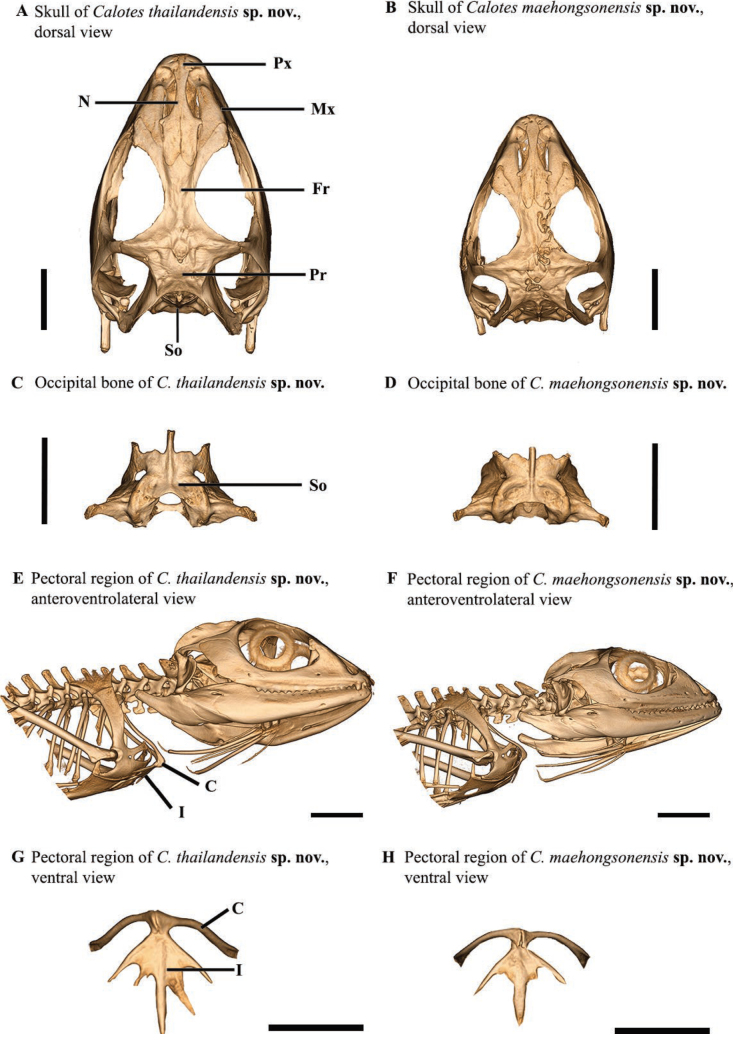
Micro-CT images of skull, occipital bone, and pectoral region of male *Calotes thailandensis* sp. nov. (**A, C**. CUMZ-R-2738 and **E, G**. CUMZ-R-2769) and male *C. maehongsonensis* sp. nov. (**B, D**. CUMZ-R-2825 and **F, H**. CUMZ-R-2820). Abbreviations: Cl, Clavicle; F, Frontal; In, Interclavicle, Mx, Maxilla; Na, Nasal; P, Parietal, Px, Premaxilla; So, Supraoccipital bones. Scale bars: 5 mm.

Osteological reconstructions of the skull and pelvic girdle regions reveal noticeable differences in shapes between *C. thailandensis* sp. nov. and *C. maehongsonensis* sp. nov. In the skull region, the anterior portion of the premaxilla of *C. thailandensis* sp. nov. is distinctively narrower compared to *C. maehongsonensis* sp. nov. (Fig. 5A, B). In addition, the supraoccipital bone, as part of the braincase, is comparatively different. In *C. thailandensis* sp. nov., the curve of the posterior portion of the supraocciptal appear to be more concave compared to that of *C. maehongsonensis* sp. nov. (Fig. 5C, D). In the pectoral region, the shapes of the interclavicle bones are remarkably differed between samples from *C. thailandensis* sp. nov. and *C. maehongsonensis* sp. nov. In *C. thailandensis* sp. nov., an extra process is observed between the lateral and posteromedian processes of the interclavicle, which is absent in *C. maehongsonensis* sp. nov., where the interclavicle resembles the typical arrow shape (Fig. 5E–H).

### Taxonomy

#### Calotes thailandensis sp. nov.

Taxon classificationAnimaliaSquamataAgamidae

AE0E7692-2E9D-5579-8DE1-7EBE3EA9E729

https://zoobank.org/A3BAF1FC-6AFD-4B38-A1BD-3379C13A7024

**Figure 6.**
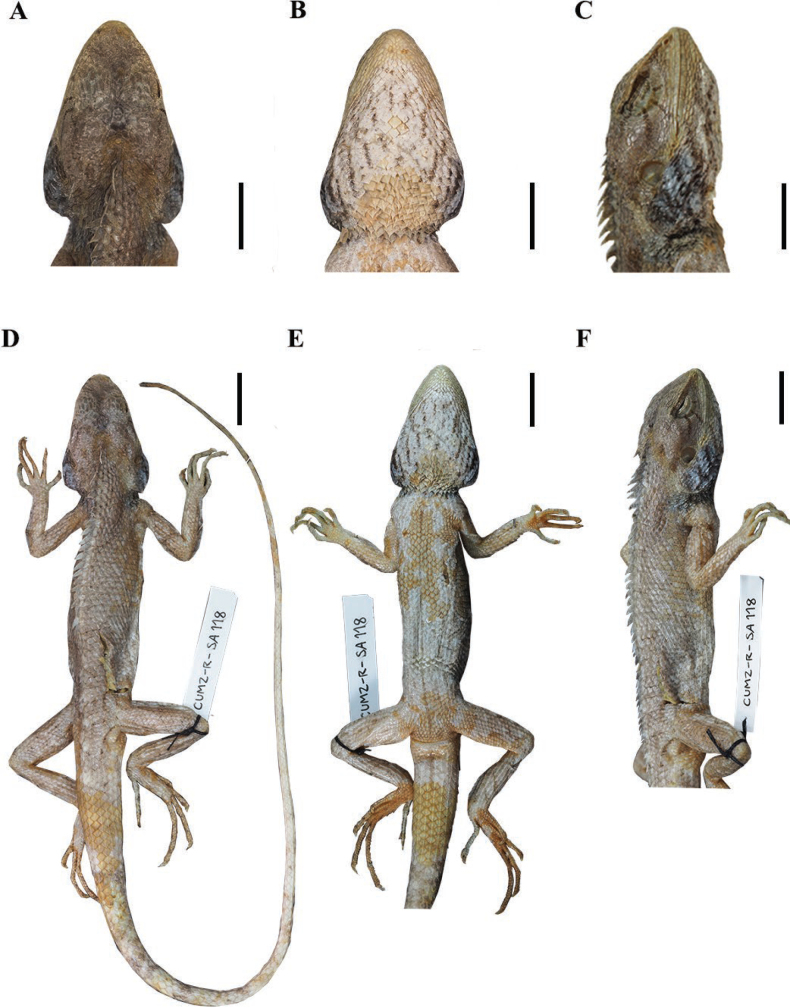
Holotype of adult male *Calotes thailandensis* sp. nov. (CUMZ-R-2738 [SA118]). **A**. Head dorsal; **B**. Head ventral; **C**. Head lateral; **D**. Full body dorsal; **E**. Body ventral, and **F**. Lateral region. Scale bars 10 mm.

**Figure 7.**
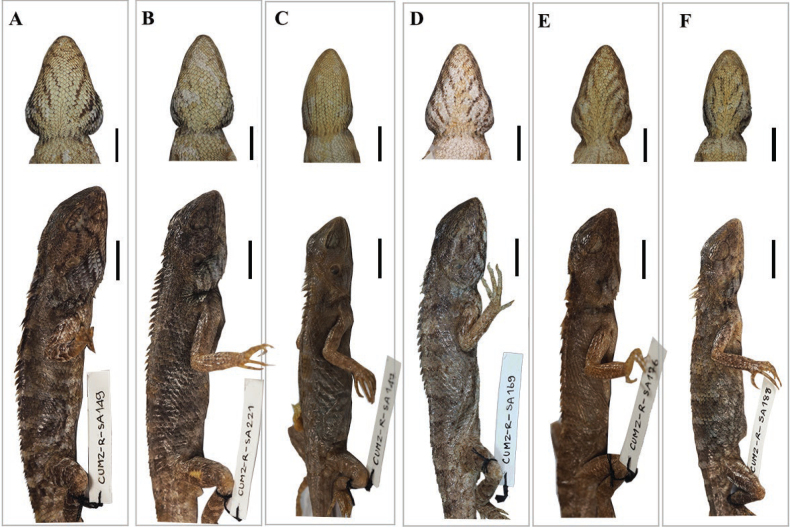
Ventral head and lateral views (**A–F**) of paratypes of *Calotes thailandensis* sp. nov. (CUMZ-R-2769[SA149], 2872[SA221], 2767[SA147]) (**A–C**) and *Calotes maehongsonensis* sp. nov. (CUMZ-R-2820[SA169], 2827[SA176], 2839[SA188]) (**D–F**) are showed consisting of adult males (**A, B, D, E**) and adult females (**C, F**). Scale bars: 10 mm.

**Figure 8.**
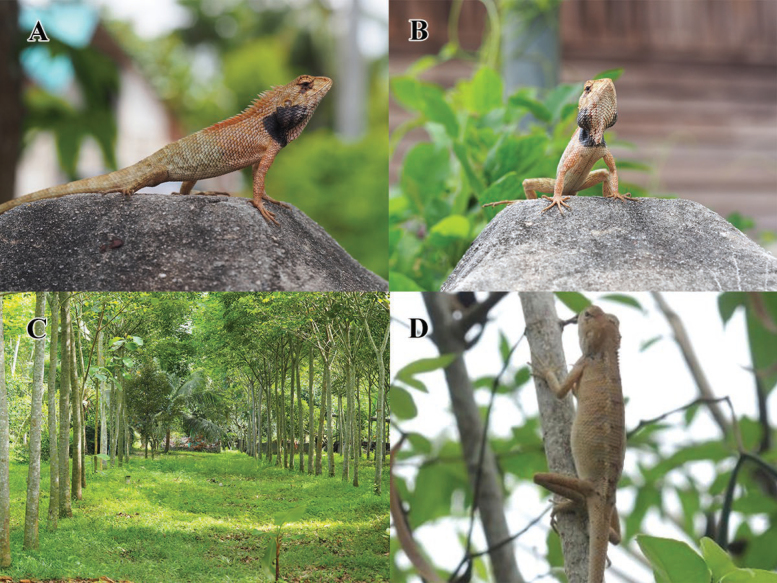
An uncollected male (**A, B**) on breeding season (March, 2025) and female (**D**) (CUMZ-R-2767) of *Calotes thailandensis* sp. nov. in rubber plantation (**C**) at Khlong Hoi Kong District, Songkhla Province, Thailand.

Figs 5, 6, 7, 8, Tables 4–6

Calotes versicolor Smith, 1935: 189 (partim); Taylor 1963: 891 (partim); Grismer 2011: 151; Chan-ard et al. 2015: 97 (partim); Prakobkarn et al. 2016: 482 (partim); Gowande et al. 2021: 680 (partim); Tantrawatpan et al. 2021: 53 (partim).
Calotes versicolor versicolor Auffenberg & Rehmann, 1993: 24 (partim); 1995: 1 (partim).

##### Type material.

***Holotype***. • CUMZ-R-2738, adult male (Fig. 6) from Khlong Hoi Kong District, Songkhla Province, Thailand (6.864937°N, 100.402773°E, 24 m a.s.l.), was collected by AP on 27 March 2009. ***Paratypes***. • CUMZ-R-2624, adult male, was collected on 30 June 2008; • CUMZ-R-2871–2872 (Fig. 7), CUMZ-R-2875, three adult males, were collected on 27 March 2009; • CUMZ-R-2769, adult male, was collected on 30 March 2009; • CUMZ-R-2767–2768 (Fig. 7), two adult females, were collected on 29 June 2008; • CUMZ-R-2846–2848, three adult females, were collected on 15 June 2009. All specimens were collected from the same locality as the holotype by AP.

##### Diagnosis.

*Calotes thailandensis* sp. nov. shares the obliquely posterior or vertical scale-row orientation on the neck and adjacent supra-axillary area with *Calotes irawadi* group. It can be separated from other congeneric species by having combination of the following characters (Tables 4, 5): 1) moderate body size (mean SVL 87.1 mm in male); 2) wide PelvW (mean 13.16 mm and range 11.62–15.07 mm) in male; 3) fewer number of Midbody (mean 42.5) and Dorsal (mean 46.0); 4) Distinct large middorsal crest spine at neck in males (~1.33 × tympanum diameter, range 1.03–1.68 × tympanum diameter) (Table 6); 5). Moderated size of supratympanic spine (~0.6 × tympanum diameter, range 0.59–0.82 × tympanum diameter) and the anterior supratympanic spine is larger than posterior supratympanic spine (Table 6, Fig. 6A); 6) hindlimb adpressed forward (HAF) reaching between tympanum and eye; 7) usual presence of dark patches on jaw muscles, presence of striping on throat, presence of continuous striping on trunk, and presence of dark stripe (like a collar) on ventral neck of male (Fig. 6B), and rarely appearance of dark stripe at ventral trunk.

**Table 6.** Spine characters of male *Calotes thailandensis* sp. nov. and male *C. maehongsonensis* sp. nov. Measurements are represented in mean ± SD (range) in millimeters (mm). Abbreviations are showed in Materials and methods.

Characters	*Calotes thailandensis* sp. nov.		*Calotes maehongsonensis* sp. nov.	
	Holotype	Paratypes	Holotype	Paratypes
	CUMZ-R-2738	Males (*n =* 5)	CUMZ-R-2825	Males (*n =* 5)
MCS	4.5	4.41 ± 0.90	3.45	4.04 ± 0.63
		(3.60–5.60)		(3.25–4.80)
ASS	2.1	2.30 ± 0.36	1.35	1.10 ± 0.11
		(2.10–2.95)		(1.00–1.25)
PSS	1.85	2.22 ± 0.17	1.2	1.14 ± 0.13
		(2.05–2.40)		(1.00–1.25)
MCS/TymD	1.34	1.33 ± 0.28	1.27	1.44 ± 0.16
		(1.03–1.68)		(1.20–1.62)
ASS/TymD	0.61	0.69 ± 0.08	0.5	0.39 ± 0.02
		(0.59–0.82)		(0.37–0.42)
PSS/TymD	0.55	0.67 ± 0.03	0.44	0.41 ± 0.06
		(0.64–0.70)		(0.33–0.47)

##### Comparisons.

*Calotes thailandensis* sp. nov. can be distinguished from true *C. irawadi* by adult males having a wider PelvW (mean 13.16 mm, 11.62–15.07 mm vs mean 8.59 mm, 6.90–10.90 mm); fewer Midbody (mean 42.5, 37–46 vs mean 45.4, 42–50) and Dorsal (mean 46, 40–49 vs mean 48.3, 41–53); a longer supratympanic spine (> 1/2 vs < 1/3 × tympanum diameter); a longer middorsal crest spine (> 1 vs ~1 × tympanum diameter); hindlimb adpressed forward (HAF) reaching between tympanum and eye vs HAF reaching tympanum in *C. irawadi*.

*Calotes thailandensis* sp. nov. can be separated from *C. maehongsonensis* sp. nov. by adult males having a wider PelvW (mean 13.16 mm, 11.62–15.07 mm vs mean 11.38 mm, 10.46–12.95 mm); greater HeadH (mean 14.18, 11.70–16.40 mm vs mean 11.70, 11.10–12.90 mm); fewer Midbody (mean 42.5, 37–46 vs mean 44.5, 43–46) and fewer Dorsal (mean 46.6, 45–49 vs mean 48.8, 47–51); longer supratympanic spine (> 0.5 vs < 0.5 × tympanum diameter); shorter HAF (HAF crossing eye in *C. maehongsonensis* sp. nov.). In osteological characters, *Calotes thailandensis* sp. nov. is distinguished from *C. maehongsonensis* sp. nov. by having 1) narrower premaxillary bone; 2) more concave on posterior portion of the supraoccipital bone; 3) presence of lateral and posteromedian processes on interclavicle bone (Fig. 5).

*Calotes thailandensis* sp. nov. can be distinguished from *C. wangi* by adult males having a smaller head size (HeadL, 19.50–23.52 mm vs 24.29–24.47 mm) and a shorter finger and toe (4FingLng, mean 10.42 mm, 9.00–11.36 mm vs 13.41–14.10 mm and 4ToeLng, mean 16.17 mm, 14.24–18.00 mm vs 19.04–21.57 mm); a greater HeadH/SVL (mean 0.16, 0.14–0.19 vs mean 0.15, 0.11–0.18) (Suppl. material 3); a longer supratympanic spine (> 0.5 vs < 0.5 × tympanum diameter).

*Calotes thailandensis* sp. nov. can be separated from *C. versicolor* (Zug et al. 2006) by adult males having a smaller body size (mean 87 mm vs mean 119 mm); more Dorsal (mean 46, 40–49 vs mean 40.8) (Table 5); a shorter middorsal crest spine (0.9–1.9 vs 2.5–3.0 × tympanum diameter) (Table 6).

*Calotes thailandensis* sp. nov. differs from *C. calotes* (Pal et al. 2018) by adult males having more Midbody (37–44 vs 30–35); a smaller supratympanic spine and a smaller middorsal crest spine.

*Calotes thailandensis* sp. nov. and *C. goetzi* share the absence of a large postorbital spine in adult males; however, *C. thailandensis* sp. nov. can be separated from *C. goetzi* by the absence of both prominent dark brown dorsolateral blotches on the dorsolateral region and a patch of small dark granular scales I front of forelimb insertion in adult males.

*Calotes thailandensis* sp. nov. differs from *C. emma* by the absence of both a large postorbital spine and a patch of small dark granular scales in front of forelimb insertion in adult males.

##### Description of holotype.

(Fig. 6): An adult male of 87.24 mm SVL, 30.38 mm SnForeL, 42.93 mm TrunkL, 13.35 mm TailH, 11.61 mm TailW, 253 mm TailL, 14.74 mm PectW, 17.26 mm UpArmL, 15.66 mm LoArmL, 14.92 mm ForefL, 10.16 mm 4FingLng, 21.86 mm UpLegL, 19.18 mm CrusL, 25.92 mm HindfL, and 16.40 mm 4ToeLng. Stunted head size: 21.26 mm HeadL, 20.18 mm HeadW, 14.21 mm JawW, 15.25 mm HeadH, 8.70 mm SnEye, 4.48 mm NarEye, 6.25 mm EyeEar, 5.27 mm SnW, and 8.60 mm InterOrb.

Head distinct from neck and triangular shape; blunt snout-tip; slightly bowed outward by jaw muscles at posterior edge of head; sides of head flat; slightly protruding eyes; flat chin and throat. Dorsally head scales vary in size and have a smooth surface; rostral equivalent to supralabials in height above lip with 7 SnS; 8/8 (left/right) elongate and sharply folded CanthR, enlarged scales in supraocular area, 13 HeadSLn and 15 HeadSTr, posteriorly slightly enlarged with bell-shaped interparietal scale. Laterally head with single large nasal scale on each side abutting rostral and perforated by large naris; small scales in loreal and preocular area; 11/10 Suplab; eye covered with small granular-sized scales, 11/11 Eyelid; postocular and temporal scales modest to small, smooth laterally and dorsolaterally; tympanum large (subequal eye-opening diameter) and naked; pair of spines or clusters in supratympanum area, anterior one dorsolaterally directly above anterior half of tympanum separated by 6 scale rows as well as posterior spine of tympanum separated by seven scale rows, a single narrow, spine-like scale (length slightly larger than 0.5 × tympanum maximum diameter) projecting upward; 10/10 IL along mouth margin; medially the chin throat scales triangular and smooth to lightly keeled; single median triangular mental scale between left and right 1^st^ supralabials and barely larger than them.

Moderately dorsally and laterally keeled trunk scalation; elongate and straight middorsal crest scales, origin separated from interparietal by 3 rows, moderate length at neck (2 × length of adjacent parasagittal scales); 46 Dorsal, 43 Midbody; keeled trunk scales, weakly scales on ventrolateral half of neck and trunk, keel and scale orientation diagonally upward from neck and supra-axillary area to base of tail, nearly vertical on anterodorsally surface of neck; modest sized and smooth preaxillary scales; large and uniform sized ventral scales from throat to vent.

Weak to modest keeled scales on limbs; bicarinate lamellae ventrally on fingers (20/21 4FingLng) and toes (22/23 4ToeLng) with 3–4 modestly keeled scales dorsally and strongly; claws long, thin, and sharply pointed on all digits.

##### Coloration of holotype.

In preservation (Fig. 6): Dorsally the head is dark yellow and ventrally whitish. There are eight brown stripes around the eyes. One stripe arises behind the eye extending unbroken to tympanum. No transvers stripe occurs on the anterior dorsal head, but diagonal stripes appear posteroventrally on head. Pale dark patch occurs on jowl muscles and continuously extended to ventral neck, looking like dark stripe. The pale brown narrow trunk bands with four bars are found, but not clearly delimited, transverse bands on above body, although there is no middorsal stripe. Discrete stripes occur on the ventral sides of the body, but forearm stripe and paired nuchal spots are absent.

##### Variation.

Variation in mensural, meristic and spine characters of the paratypes of *Calotes thailandensis* sp. nov. are presented in Tables 4–6. Those characters are congruent with the holotype, excluded in coloration pattern (Figs 6, 8). Coloration of paratypes in preservation. In adult male paratypes, transverse bands vary from pale brown narrow to dark brown board bands, with straight lines lying on left and right sides, whereas transverse bands dorsally on females are usual present in both straight and zig-zags. Coloration in life (Fig. 7). Pale brown transverse bands are usually present on dorsal body of most samples in both sexes.

##### Distribution.

*Calotes thailandensis* sp. nov. has the broad distribution on southern, central, eastern, western, and northeastern Thailand (Fig. 1). It is represented by vouchers from southern Thailand collected at Songkhla, Krabi and Ranong Province as well as vouchers from central Thailand collected at Saraburi Province. The distribution range of this species likely extends beyond the Thailand-Malaysia border into Peninsular Malaysia and Singapore which was previously reported as *C. versicolor* by Grismer (2011).

##### Habitat and natural history notes.

This species inhabits agricultural areas (such as rubber plantation) (Fig. 8A) and man-made habitat (Fig. 8B, C). Male samples were usually found on tree trunk, or fence row, whereas female samples were often hidden within the bushes or the small trees during the day. Gravid females were collected during March-May suggesting the breeding season coincides with the early rainy season,

##### Etymology.

The specific epithet *thailandensis* is selected because of this species wide occurrence throughout most of Thailand, except northern and northwestern Thailand.

#### Calotes maehongsonensis sp. nov.

Taxon classificationAnimaliaSquamataAgamidae

D595BEB9-91E1-5B2E-A03F-48844D6B95B7

https://zoobank.org/260DF0B5-A546-4054-B23E-8C33E962B86A

**Figure 9.**
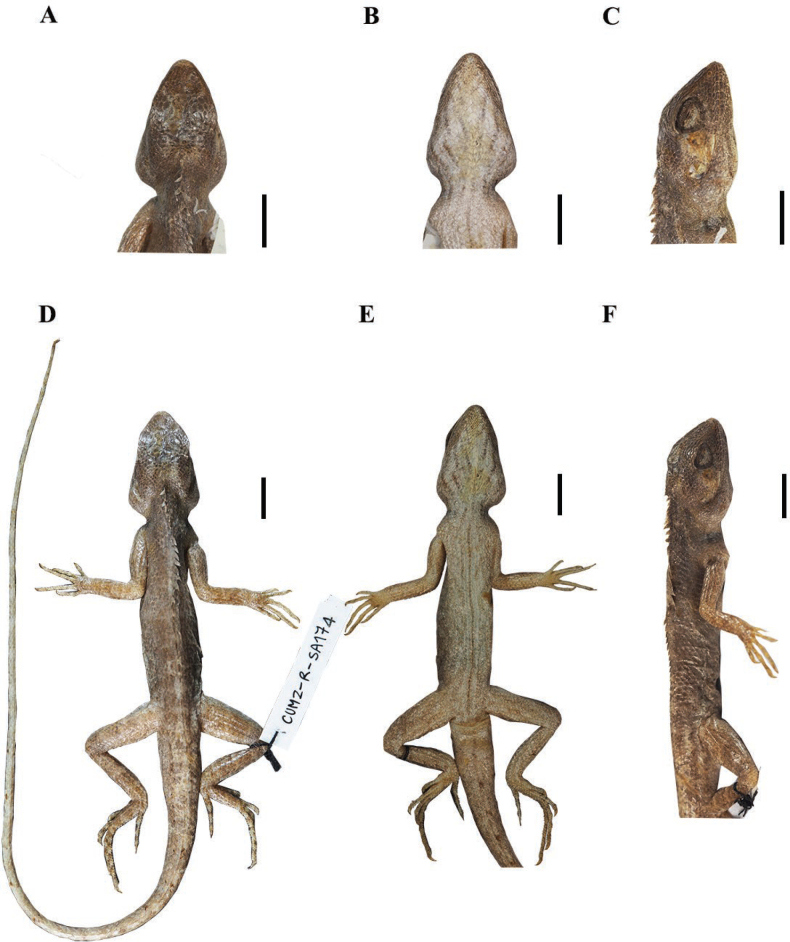
Holotype of adult male *Calotes maehongsonensis* sp. nov. (CUMZ-R-2825[SA174]). **A**. Head dorsal; **B**. Head ventral; **C**. Head lateral; **D**. Full body dorsal; **E**. Body ventral, and **F**. Lateral region. Scale bars: 10 mm.

**Figure 10.**
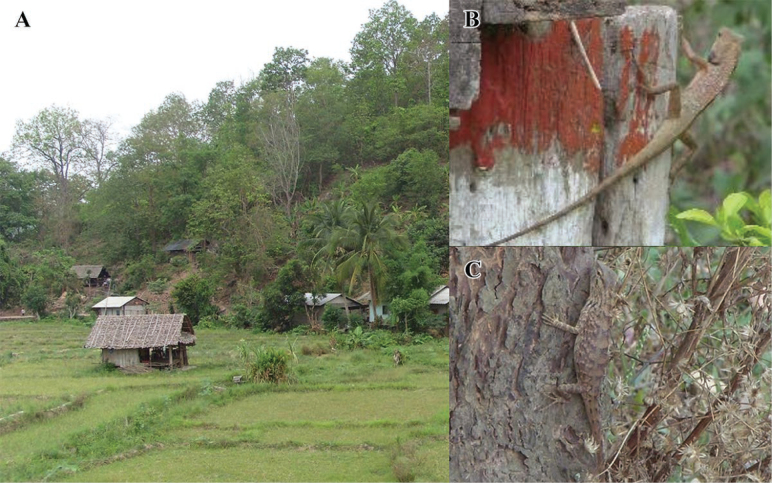
Habitat (**A**) of the type locality and male (**B**) (CUMZ-R-2820) and female (**C**) (CUMZ-R-2839) of *Calotes maehongsonensis* sp. nov. at Khun Yuam District, Mae Hong Son Province, Thailand.

Figs 5, 7, 9, 10, Tables 4–6

Calotes versicolor Smith, 1935: 189 (partim); Taylor 1963: 891 (partim); Chan-ard et al. 2015: 97 (partim); Prakobkarn et al. 2016: 482 (partim); Gowande et al. 2021: 680 (partim); Tantrawatpan et al. 2021: 53 (partim).
Calotes versicolor versicolor Auffenberg & Rehman, 1993: 24 (partim); 1995: 1 (partim).

##### Type material.

***Holotype***. • Adult male (CUMZ-R-2825) (Fig. 9) from Khun Yuam District, Mae Hong Son Province, Thailand (18.801636°N, 97.898188°E, 499 m a.s.l.), collected by AP on 09 May 2009. ***Paratypes***. • Five adult males (CUMZ-R-2820, CUMZ-R-2823, CUMZ-R-2827–2828, and CUMZ-R-2832 and five adult females (CUMZ-R-2833, 2835–2837, and 2839) (Fig. 7, Tables 4–6) were collected on 9 May 2009, which is the same collection date as the holotype. All samples were also collected at the same locality as the holotype.

##### Diagnosis.

*Calotes maehongsonensis* sp. nov. shares the obliquely posterior or vertical scale-row orientation on the neck and adjacent supra-axillary area with *C. irawadi* and *C. thailandensis* sp. nov.. It differs from other congeneric species by the combination of the following characters: 1) having smaller head size (HeadH, JawW, SnN, and SnForeL); 2) more Midbody (mean 44.5, range 43–46) and Dorsal (mean 48.8, range 47–51); 3) moderate middorsal crest spine length (mean 1.44 × tympanum diameter, range 1.20–1.62 × tympanum diameter); 4) short supratympanic spine (< 0.5 × tympanum diameter) (Table 6); 5) longer hindlimb adpressed forward (HAF) crossing eye.

##### Comparisons.

*Calotes maehongsonensis* sp. nov. can be distinguished from true *C. irawadi* by adult males having a smaller head size (smaller HeadH, JawW, SnW and SnForel) (Fig. 4); a wider PelvW (mean 11.38 mm, 10.46–12.95 mm vs mean 8.59 mm, 6.90–10.90 mm); a longer middorsal crest spine (>1 vs ~1 × tympanum diameter); HAF crossing eye vs HAF reaching tympanum in *C. irawadi*. It also differs from *C. irawadi* by the usual presence of dark patch in jaw muscle and presence of colored patch on throat.

*Calotes maehongsonensis* sp. nov. differs from *C. thailandensis* sp. nov. by adult males having a smaller head size (smaller headH, JawW, SnW and SnForel) (Suppl. material 5: fig. S1); a smaller PelvW (mean 11.38 mm, 10.46–12.95 mm vs mean 13.16 mm, 11.62–15.07 mm) (Table 4); more Midbody (mean 44.5, 43–46 vs mean 42.5, 37–46) and more Dorsal (mean 48.8, 47–51 vs mean 46, 40–49) (Table 5, Suppl. material 5: fig. S2); a shorter supratympanic spine ((<0.5 vs > 0.5 × tympanum diameter) and the posterior supratympanic spine slightly equal to anterior supratympanic spine (Table 6); hindlimb adpressed forward (HAF) crossing eye (HAF reaching between tympanum and eye in *C. thailandensis* sp. nov.). In osteological characters, *C. maehongsonensis* sp. nov. is distinguished from *C. thailandensis* sp. nov. by having 1) Blunt premaxillary bone; 2) Less concave on posterior portion of the supraoccipital bone; 3) Absence of lateral and posteromedian processes on interclavicle bone (Fig. 5). In coloration, *C. maehongsonensis* sp. nov. lacks a dark collar-like stripe on ventral neck of males, but dark collar-like stripe occurs ventrally on neck of male *C. thailandensis* sp. nov. (Fig. 7).

*Calotes maehongsonensis* sp. nov. can be distinguished from *C. wangi* by adult males having a smaller body size (SVL, 76.16–83.38 mm vs 88.34–96.4 mm), a smaller head size (EyeEar, InterOrb, HeadH, JawW, SnW, HeadL, SnForeL), and shorter finger and toe (4FingLng: mean 9.48 mm, 8.80–10.46 mm vs 13.41–14.10 mm; 4ToeLng: mean 14.34 mm, 13.33–15.89 mm vs 19.04–21.57 mm) (Table 4); a greater HeadH/SVL (mean 0.16, 0.14–0.19 vs mean 0.15, 0.11–0.18) (Suppl. material 3); HAF crossing the eye vs HAF reaching between tympanum and eye in *C. wangi*.

*Calotes maehongsonensis* sp. nov. differs from *C. versicolor* (Zug et al. 2006) by adult males having a smaller body size (mean 78 mm vs mean 119 mm); more Midbody (mean 44.5, 43–46 vs mean 42.8); more Dorsal (mean 48.8, 47–51 vs mean 40.8); a shorter middorsal crest spine (1.2–1.8 vs 2.5–3.0 × tympanum diameter) (Table 6).

*Calotes maehongsonensis* sp. nov. differs from *C. calotes* (Pal et al. 2018) by adult males having more Midbody (43–46 vs 30–35); a shorter supratympanic spine and a shorter middorsal crest spine.

*Calotes maehongsonensis* sp. nov. can be separated from *C. goetzi* by the absence of both prominent dark brown dorsolateral blotches and a path of small dark granular scales in front of forelimb insertion in adult males.

*Calotes maehongsonensis* sp. nov. differs from *C. emma* by adult males lacking large postorbital spine and patch of small dark granular scales in front of forelimb insertion.

##### Description of holotype.

(Fig. 9): An adult male of 76.16 mm SVL, 23.72 mm SnForeL, 34.54 mm TrunkL, 10.72 mm TailH, 9.37 mm TailW, 210 mm TailL, 13.38 mm PectW, 13.47 mm UpArmL, 12.03 mm LoArmL, 13.15 mm ForefL, 8.80 mm 4FingLng, 19.20 mm UpLegL, 16.72 mm CrusL, 21.02 mm HindfL, and 13.08 mm 4ToeLng. Head pentagonal (dorsal outline) covered largely with small, mostly smooth scales slightly overlapping; 23.00 mm HeadL, 18.68 mm HeadW, 12.24 mm JawW, 11.66 mm HeadH, 7.55 mm SnEye, 4.34 mm NarEye, 4.80 mm EyeEar, 4.12 mm SnW, and 7.42 mm InterOrb.

Head distinct from neck; triangle head with blunt snout-tip; sides of head flat, sharp canthus rostralis and supraciliary edge to lips with slightly rotund eye; slightly protruding eye; generally flat chin and throat. Dorsally head scales are smooth and vary in size and scales of some individuals are equal to trunk scales. Rostral scale size is equal to supralabials in height; 5 SnS; 7/8 (left/right) elongate and sharply folded CanthR, enlarged scales on supraocular area, 14 HeadSLn and 13 HeadSTr; irregularly shaped interparietal scales. Laterally head with single large nasal scale; small scales at loreal and preocular area with two parallel longitudinal rows above supralabials; 11/11 SL; double row of eyelid scales with outer row pyramidal-like scales, inner row smooth and flat 13/14 Eyelid; modest to small and smooth postocular and temporal scales; large and naked tympanum; pair of tympanic spines in supratympanic area are separated by seven scale rows; posterior supratympanic spine is a single narrow, spine-like scale (length slightly < 0.5 × tympanum maximum diameter) projecting upward; 10/10 IL along mouth margin; first supralabials are barely larger than other supralabials.

Keeled trunk scales dorsally and laterally; elongated middorsal crest scale (3 × length of adjacent parasagittal scales) separated from interparietal by four rows; 51 Dorsal, 44 Midbody.

Modest to large and keeled scales on limbs; strongly bicarinate lamellae ventrally of fingers (19/19 4FingLng) and toes (23/23 4ToeLng); long and thin claws and sharply pointed on all digits.

##### Coloration of holotype.

In preservation (Fig. 9). Dorsally the head is brown and ventrally whitish. There are six pale brown stripes around the eyes. No transverse stripe occurs on the anterior dorsal head, but diagonal stripes appear on ventral posterior head. There is pale dark patch on jowl muscles, but absence of dark patch on ventral neck; trunk transverse bands and middorsal stripe on above body are also absence. Distinct stripes occur on the ventral sides of the body, but absence of forearm stripe and pairs of nuchal spots.

##### Variation.

Variation in morphometry and meristic characters, spine characters, and stripe pattern of paratype specimens are shown in Fig. 7 and Tables 4–6. Those characters are congruent with the holotype, excluded in coloration pattern. Coloration of paratypes in preservation, in adult male paratypes, transverse bands are rarely found, although broad transverse bands are usually present and straight in males and straight or zig-zag in females. Coloration in life (Fig. 10). Pale brown transverse bands on dorsal surface of body are usually absent in male and usually present in female.

##### Distribution.

*Calotes maehongsonensis* sp. nov. has the restricted occurrence in western and northern Thailand (Kanchanaburi and Mae Hong Son Provinces) along Thailand-Myanmar border and southeastern Myanmar including Mon, Bago, and Yangon (Fig. 1).

##### Habitat and natural history notes.

Its name is represented by type locality from Mae Hong Son Province, Northern Thailand (at approx. more than 500 m elevation). This species inhabits forest edges, primarily occurring on high hills but also adapting to anthropogenic habitats including disturbed secondary forests, agricultural areas, and rural settlements (Fig. 10). Specimens were typically observed on tree trunks and branches 1–4 meters above ground, often basking in sunlight during morning hours. The species appears to be insectivorous, with observed prey including ants and small millipede.

##### Etymology.

The specific epithet *maehongsonensis* is referred from its type locality in Mae Hong Son Province (Fig. 10), and they were also found in Kanchanaburi Province, Thailand and Southern Myanmar.

## Discussion

Using an integrative approach based on morphology and molecular analyses, we clarified the systematics of the *C. irawadi* complex in Thailand and described two new species: *C. thailandensis* sp. nov. and *C. maehongsonensis* sp. nov., occurring in different geographical regions. Substantial genetic distances (4.03–7.08% for ND2; 3.72–5.36% for COI gene) exist between these two new species and their closely related species, *C. wangi* and *C. irawadi*, that are comparable to other taxonomic studies on *Calotes* species (4.6–5.6% between *C. wangi* and *C. irawadi* in Huang et al. 2023; 4–7% between *C. mystaceus* and *C. goetzi* in Wagner et al. 2021). *Calotes thailandensis* sp. nov. is distributed across central, eastern, northeastern, and southern Thailand, while *C. maehongsonensis* sp. nov. occurs in western and northwestern Thailand and extends into parts of southern Myanmar (Fig. 1). Although most northeastern populations were described as *C. thailandensis* sp. nov., the identity of some populations in northern and northeastern Thailand, including along both sides of the Mekong River, remains uncertain owing to the absence of morphology and genetic evidence (Tantrawatpan et al. 2021; Prakobkarn et al. 2024). Recently, the revised checklist of reptiles of Indochina by Poyarkov et al. (2023) revealed the extended distribution of *C. irawadi* in northwestern Thailand. The results of this study suggested that the putative *C. irawadi* that was reported in northwestern Thailand (Poyarkov et al. 2023) was actually *C. maehongsonensis* sp. nov. In this study, none of the sequences from Thai samples was embedded within true *C. irawadi*; therefore, the occurrence of the true *C. irawadi* in Thailand remains suspicious. The taxonomic status of these Thai populations should be further studied.

Notably, this study reveals several diagnostic characters in *C. thailandensis* sp. nov. and *C. maehongsonensis* sp. nov., most importantly: (1) the hindlimb adpressed forward (HAF) reaching the orbit, or between the tympanum and orbit; and (2) a dark (like-collar) stripe on ventral surface of the neck. Besides external morphology, additional osteological features, composed of skull and interclavicle, seem to be diagnostic characters in other lizard groups with complex species relationships such as *Kolekanos* (Lobón-Rovira et al. 2022), *Tympanocryptis* (Melville et al. 2019), and *Geckolepis* (Scherz et al. 2017). Moreover, this study is the first to apply osteological characters as diagnostic features in the taxonomy of *Calotes*.

Our findings increase the number of *Calotes* species occurring in Thailand to four: *C. emma* Gray, 1845, *C. goetzi* Wagner, Ihlow, Hartmann, Flecks, Schmitz & Böhme, 2021, *C. thailandensis* sp. nov., and *C. maehongsonensis* sp. nov. Furthermore, this study also confirms that true *Calotes irawadi* and true *C. versicolor* are absent from Thailand. Based on Zug et al. (2006), Wagner et al. (2021), and the finding of this study, we confirm that neither *C. mystaceus* nor *C. htunwini* occurs in Thailand either. *Calotes htunwini* is restricted to the central dry zone of Myanmar with no specimens recorded in Thailand to date. Previous Thai records of *C. mystaceus* were revised and reassigned to *C. goetzi* by Wagner et al. (2021). Our phylogenetic analyses also clarify the taxonomic status of the Moyingyi population in Myanmar reported by Zug et al. (2006): the Moyingyi specimen from Bago Region (GenBank accession no. DQ289471) is nested within the *C. maehongsonensis* sp. nov. clade. However, the taxonomic status of some *Calotes* populations along the Mekong River in Thailand and Laos PDR, previously investigated by Tantrawatpan et al. (2021), remain uncertain. Subsequently, the taxonomic status of those populations in Thailand should be resolved with further studies.

### Key to *Calotes* species in Thailand

**Table:** 

1	Presence of a patch of small dark granular scales in front of forelimb insertion	**2**
–	Absence of both a patch of small dark granular scales in front of forelimb insertion and a large postorbital spine	**3**
2	Presence of a large postorbital spine	***C. emma***
–	Absence of a large postorbital spine, but presence of prominent dark-brown dorsolateral blotches	***C. goetzi***
3	Absence of dark (collar-like) stripe on ventral surface of neck; hindlimbs adpressed forward (HAF) crossing orbit	***C. maehongsonensis* sp. nov**.
–	Presence of dark (collar-like) stripe on ventral surface of neck, distinct during breeding season; hindlimbs adpressed forward (HAF) reach between tympanum and orbit	***C. thailandensis* sp. nov**.

## Supplementary Material

XML Treatment for Calotes thailandensis

XML Treatment for Calotes maehongsonensis

## Additional information

### Artificial Intelligence (AI) use

The authors accept full responsibility for the content of the manuscript, including the disclosure of any use of AI.

No AI tools were used in the preparation of this manuscript.

### Author ORCIDs

A. Prakobkarn https://orcid.org/0009-0000-9047-1102

G.R. Zug https://orcid.org/0000-0003-4922-5721

T. Ngamprasertwong https://orcid.org/0000-0003-3146-1849

### Data availability

All of the data that support the findings of this study are available in the main text or Supplementary Information.

## Supplementary materials

Supplementary material 1 Supplementary information 1 Data type xlsx Explanation note Summary of principal component analysis score for 25 mensural characters in Calotes irawadi complex from central Myanmar (*Calotes irawadi*) (28 males; 14 females), central and southern Thailand (*C. thailandensis* sp. nov.) (26 males; 27 females) and Mae Hong Son, northwestern Thailand (*C. maehongsonensis* sp. nov.) (10 males; 10 females). Abbreviations are listed in the Materials and methods. https://binary.pensoft.net/file/1666617This dataset is made available under the Open Database License (http://opendatacommons.org/licenses/odbl/1.0/). The Open Database License (ODbL) is a license agreement intended to allow users to freely share, modify, and use this Dataset while maintaining this same freedom for others, provided that the original source and author(s) are credited. Arpapan Prakobkarn, George R. Zug, Nontivich Tandavanitj, Thongchai Ngamprasertwong

Supplementary material 2 Supplementary information 2 Data type xlsx Explanation note Summary of principal component analysis score for 11 meristic characters in *Calotes irawadi* complex from central Myanmar (*C. irawadi*) (28 males; 14 females), central and southern Thailand (*C. thailandensis* sp. nov.) (26 males; 27 females) and Mae Hong Son, northwestern Thailand (*C. maehongsonensis* sp. nov.) (10 males; 10 females). Abbreviations are listed in the Materials and methods. https://binary.pensoft.net/file/1666618This dataset is made available under the Open Database License (http://opendatacommons.org/licenses/odbl/1.0/). The Open Database License (ODbL) is a license agreement intended to allow users to freely share, modify, and use this Dataset while maintaining this same freedom for others, provided that the original source and author(s) are credited. Arpapan Prakobkarn, George R. Zug, Nontivich Tandavanitj, Thongchai Ngamprasertwong

Supplementary material 3 Comparison of mensural character per SVL between two new species and other species in *C. irawadi* complex Data type xlsx https://binary.pensoft.net/file/1666619This dataset is made available under the Open Database License (http://opendatacommons.org/licenses/odbl/1.0/). The Open Database License (ODbL) is a license agreement intended to allow users to freely share, modify, and use this Dataset while maintaining this same freedom for others, provided that the original source and author(s) are credited. Arpapan Prakobkarn, George R. Zug, Nontivich Tandavanitj, Thongchai Ngamprasertwong

Supplementary material 4 Supplementary information 4 Data type xlsx Explanation note Summary of univariate analysis of non-meristic characters (spine length) in male *Calotes thailandensis* sp. nov. from central and southern Thailand and *C. maehongsonensis* sp. nov. from Mae Hong Son, northwestern Thailand. Data are shown as Mean ± SD in millimeter and in ratio (length per tympanum diameter; TD), with ranges. https://binary.pensoft.net/file/1666620This dataset is made available under the Open Database License (http://opendatacommons.org/licenses/odbl/1.0/). The Open Database License (ODbL) is a license agreement intended to allow users to freely share, modify, and use this Dataset while maintaining this same freedom for others, provided that the original source and author(s) are credited. Arpapan Prakobkarn, George R. Zug, Nontivich Tandavanitj, Thongchai Ngamprasertwong

Supplementary material 5 Supplementary information 5 Data type zip Explanation note **figure S1**. Box plots of the adjusted-morphological characters having statistically significant mean differences among male *Calotes irawadi* (*n* = 28), *C. thailandensis* sp. nov. (*n* = 26) and *C. maehongsonensis* sp. nov. (*n* = 10). Colored boxes represent the interquartile range, the black horizontal bar is the median, and the white spot is the mean. *, **, and *** denotes characters with significantly different mean values at *p* < 0.05, *p* < 0.01 and *p* < 0.001, respectively. Data were tested by ANOVA with post hoc test for parametric data and by Kruskal–Wallis with Dunn’s test for non-parametric data. **figure S2**. Histograms show the rang of scale among males of *C. irawadi* (*n* = 28) from Central Myanmar as well as populations of *C. thailandensis* sp. nov. (*n* = 26) and *C. maehongsonensis* sp. nov. (*n* = 10) from Thailand. Solid, dashed, dotted lines are represented by the mean values of each group. *, ** and *** denotes characters with significantly different mean values at *p* < 0.05, *p* < 0.01 and *p* < 0.001, respectively. Data were tested by Kruskal–Wallis with Dunn’s test for non-parametric data. **figure S3**. Box plots of the adjusted-morphological characters having statistically significant mean differences among female *Calotes irawadi* (*n* = 14), *C. thailandensis* sp. nov. (*n* = 27) and *C. maehongsonensis* sp. nov. (*n* = 10). Colored boxes represent the interquartile range, the black horizontal bar is the median, and the white spot is the mean. *, ** and *** denote characters with significantly different mean values at *p* < 0.05, *p* < 0.01 and *p* < 0.001, respectively. Data were tested by ANOVA with post hoc test for parametric data and by Kruskal–Wallis with Dunn’s test for non-parametric data. **figure S4**. Histograms show the rang of scale among female *C. irawadi* (*n* = 14) from Central Myanmar as well as female *C. thailandensis* sp. nov. (*n =* 27) and *C. maehongsonensis* sp. nov. (*n* = 10) from Thailand. Solid, dashed, dotted lines are represented by the mean values of each group. *** denote characters with significantly different mean values at *p* < 0.001, respectively. Data were tested by Kruskal–Wallis with Dunn’s test for non-parametric data. https://binary.pensoft.net/file/1666621This dataset is made available under the Open Database License (http://opendatacommons.org/licenses/odbl/1.0/). The Open Database License (ODbL) is a license agreement intended to allow users to freely share, modify, and use this Dataset while maintaining this same freedom for others, provided that the original source and author(s) are credited. Arpapan Prakobkarn, George R. Zug, Nontivich Tandavanitj, Thongchai Ngamprasertwong
