# Supplementary material for: Systematics of the Calotes irawadi complex (Squamata, Agamidae) with two newly described species from Thailand
Source: Zookeys. 2026 Jun 3;1281:69–104. doi: 10.3897/zookeys.1281.175455 (PMC13254549; doi:10.3897/zookeys.1281.175455)
Supplement: Supplementary material 5 — Supplementary information 5 [file zookeys-1281-069_article-175455__-s005.zip › 175455_1C-1-A_revised_supplementary_material_5.docx]

**Supplementary materials**


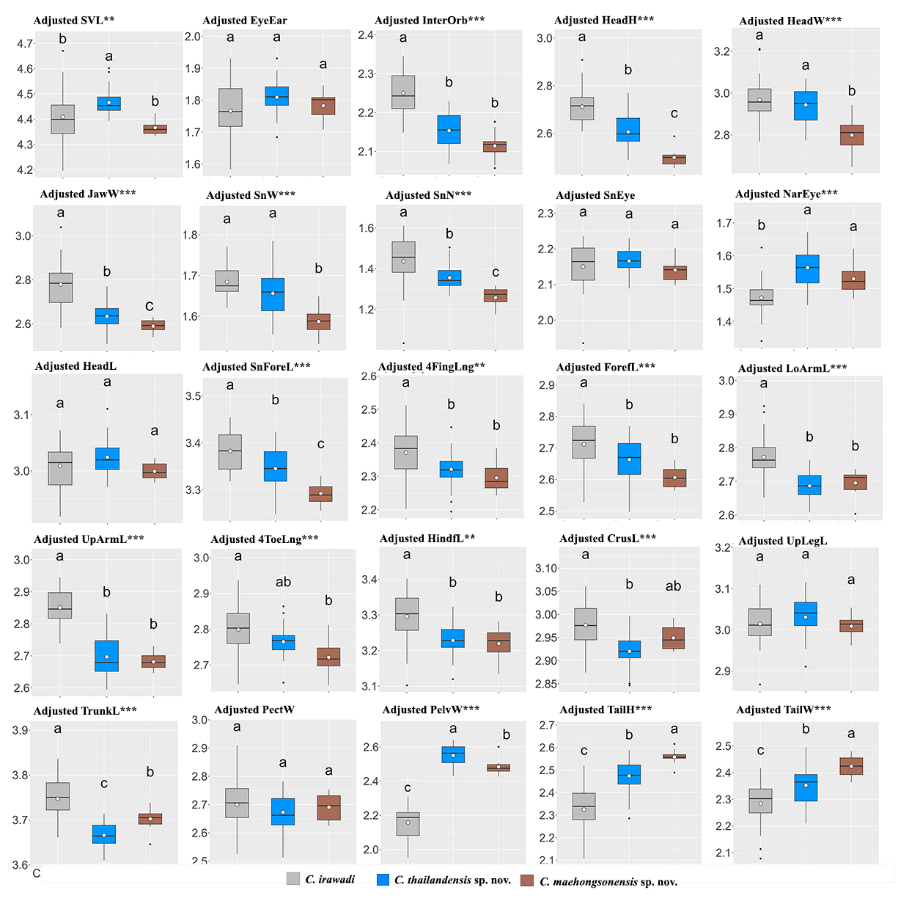


**Supplementary material 5**

**Figure S1.** Box plots of the adjusted-morphological characters having statistically significant mean differences among male *Calotes irawadi* (*n* = 28), *C. thailandensis* **sp. nov.** (*n* = 26) and *C. maehongsonensis* **sp. nov.** (*n* = 10). Colored boxes represent the interquartile range, the black horizontal bar is the median, and the white spot is the mean. *, ** and *** denotes characters with significantly different mean values at *p* < 0.05, *p* < 0.01 and *p* < 0.001, respectively. Data were tested by ANOVA with post hoc test for parametric data and by Kruskal−Wallis with Dunn’s test for non-parametric data.


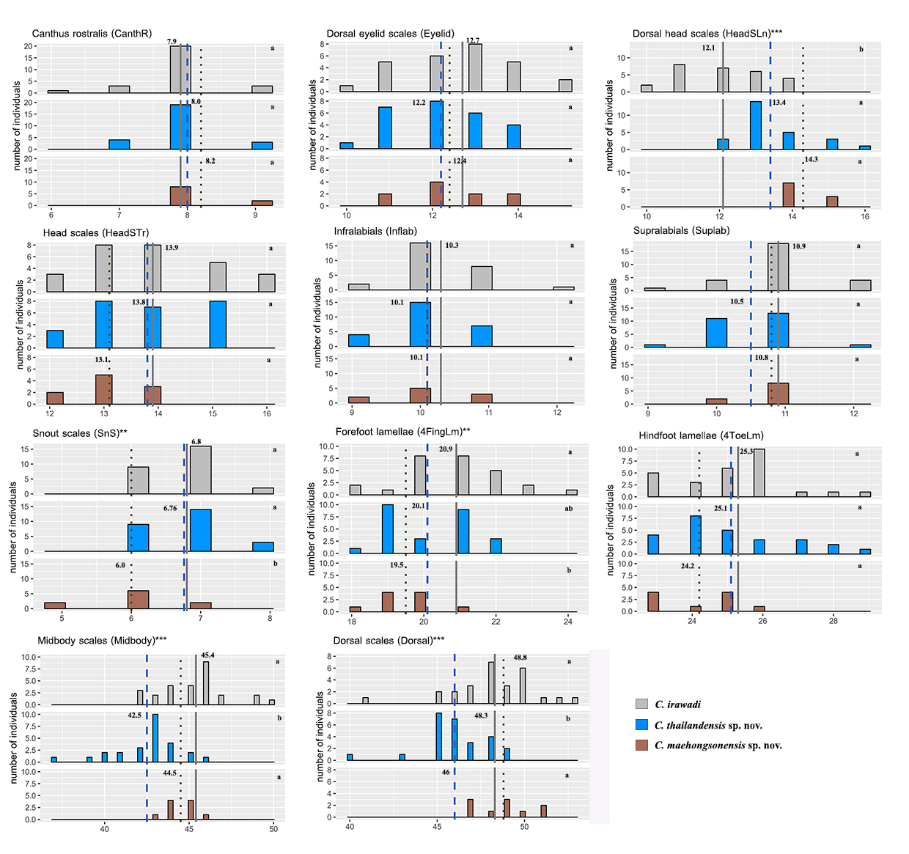


**Supplementary material 6**

**Figure S2.** Histograms show the rang of scale among males of *C. irawadi* (*n* = 28) from Central Myanmar as well as populations of *C. thailandensis* **sp. nov.** (*n* = 26) and *C. maehongsonensis* **sp. nov.** (*n* = 10) from Thailand. Solid, dashed, dotted lines are represented by the mean values of each group. *, ** and *** denotes characters with significantly different mean values at *p* < 0.05, *p* < 0.01 and *p* < 0.001, respectively. Data were tested by Kruskal–Wallis with Dunn’s test for non-parametric data.


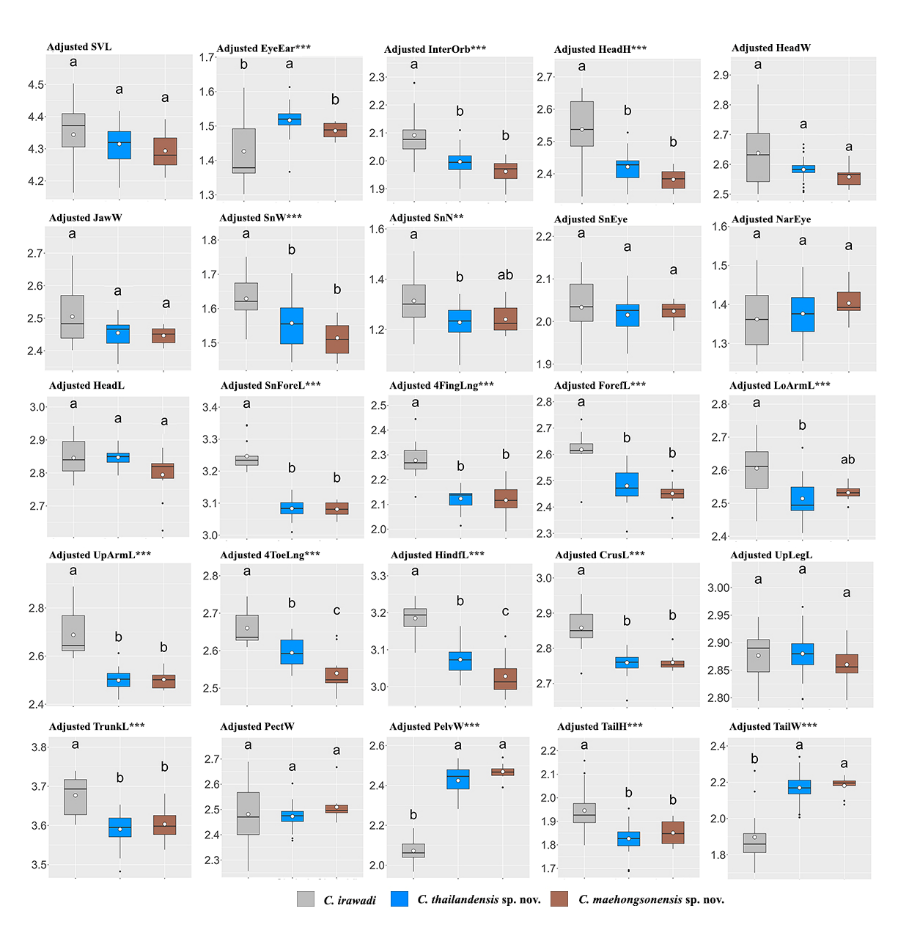


**Supplementary material 7**

**Figure S3.** Histograms show the rang of scale among males of *C. irawadi* (*n =* 28) from Central Myanmar as well as populations of *C. thailandensis* sp. nov. (*n =* 26) and *C. maehongsonensis* sp. nov. (*n =* 10) from Thailand. Solid, dashed, dotted lines are representhe white spot is the mean. *, ** and *** denote characters with significantly different mean values at *p* < 0.05, *p* < 0.01 and *p* < 0.001, respectively. Data were tested by ANOVA with post hoc test for parametric data and by Kruskal–Wallis with Dunn’s test for non-parametric data.


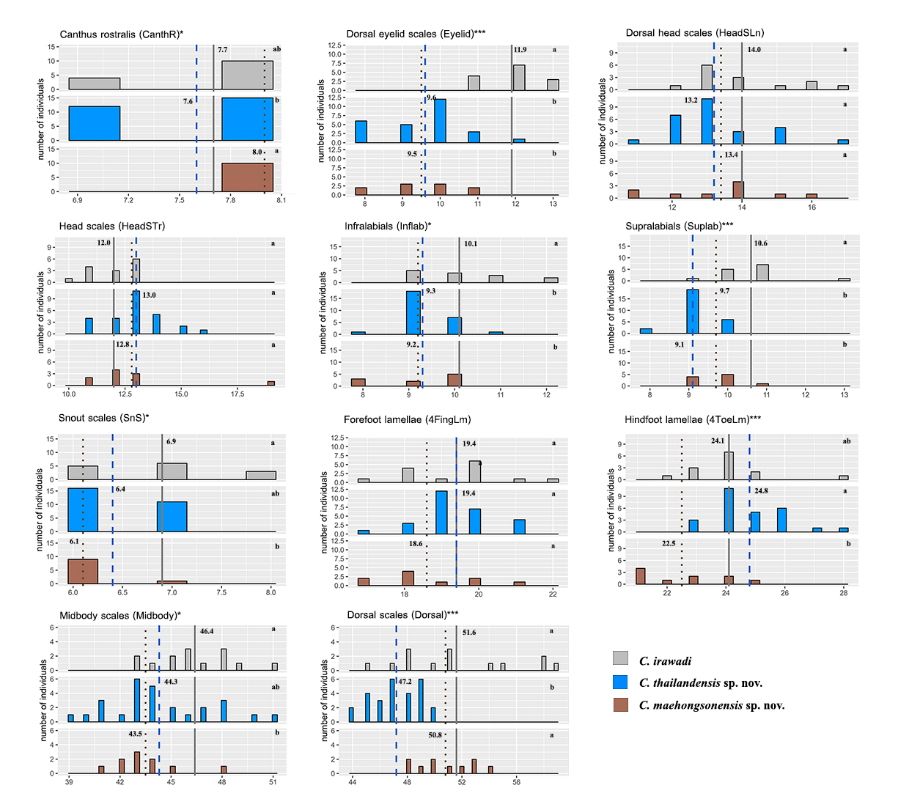


**Supplementary material 8**

**Figure S4.** Histograms show the rang of scale among female *C. irawadi* (*n* = 14) from Central Myanmar as well as female *C. thailandensis* **sp. nov.** (*n* = 27) and *C. maehongsonensis* **sp. nov.** (*n* = 10) from Thailand. Solid, dashed, dotted lines are represented by the mean values of each group. *** denote characters with significantly different mean values at *p* < 0.001, respectively. Data were tested by Kruskal–Wallis with Dunn’s test for non-parametric data.
