# Supplementary figures and images for: Systematics of the Calotes irawadi complex (Squamata, Agamidae) with two newly described species from Thailand
Source: Zookeys. 2026 Jun 3;1281:69–104. doi: 10.3897/zookeys.1281.175455 (PMC13254549; doi:10.3897/zookeys.1281.175455)

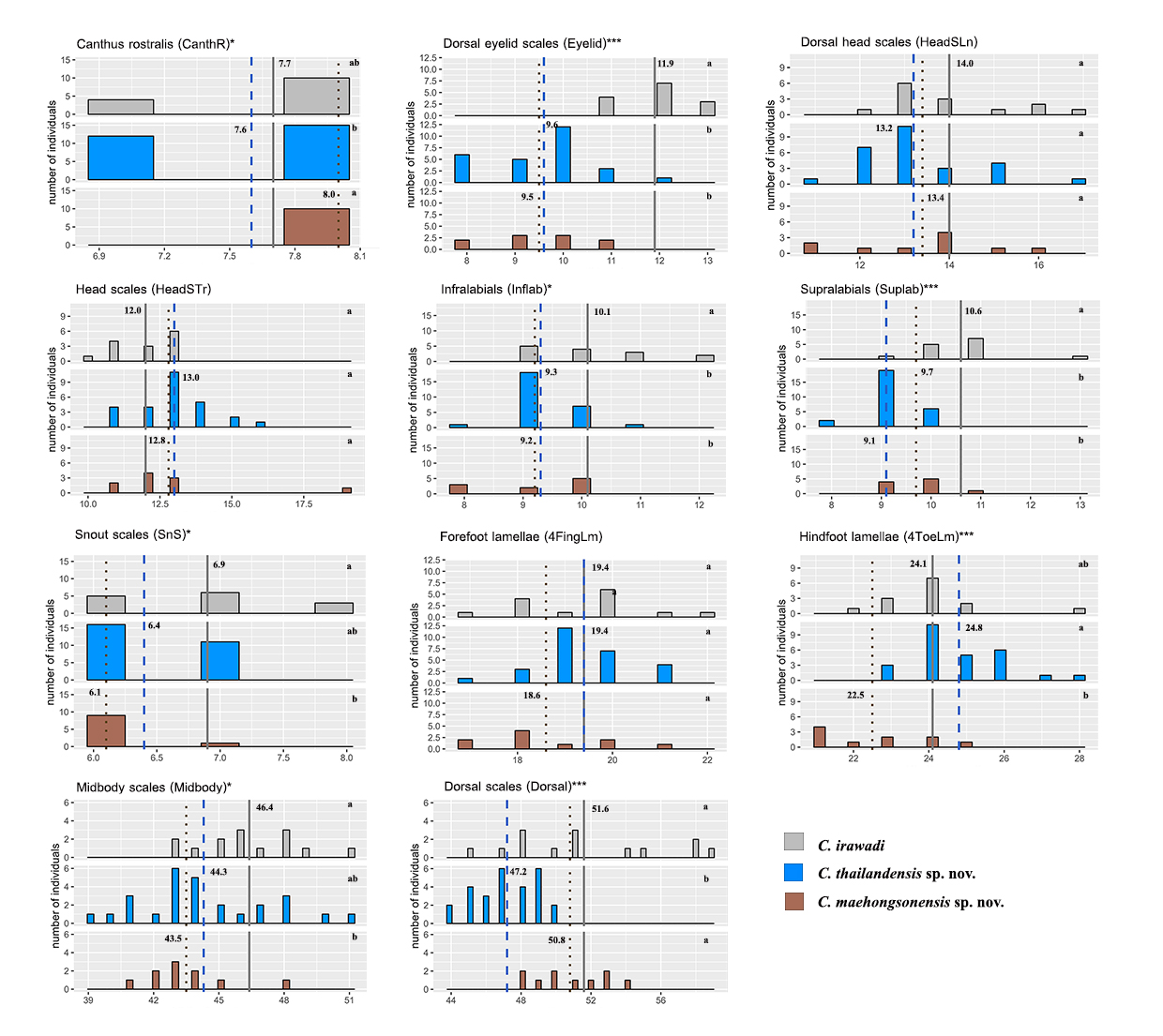

Supplement: Supplementary material 5 — Supplementary information 5 [file zookeys-1281-069_article-175455__-s005.zip › 175455_1C-1-A_supplementary_Figure_4.tif]

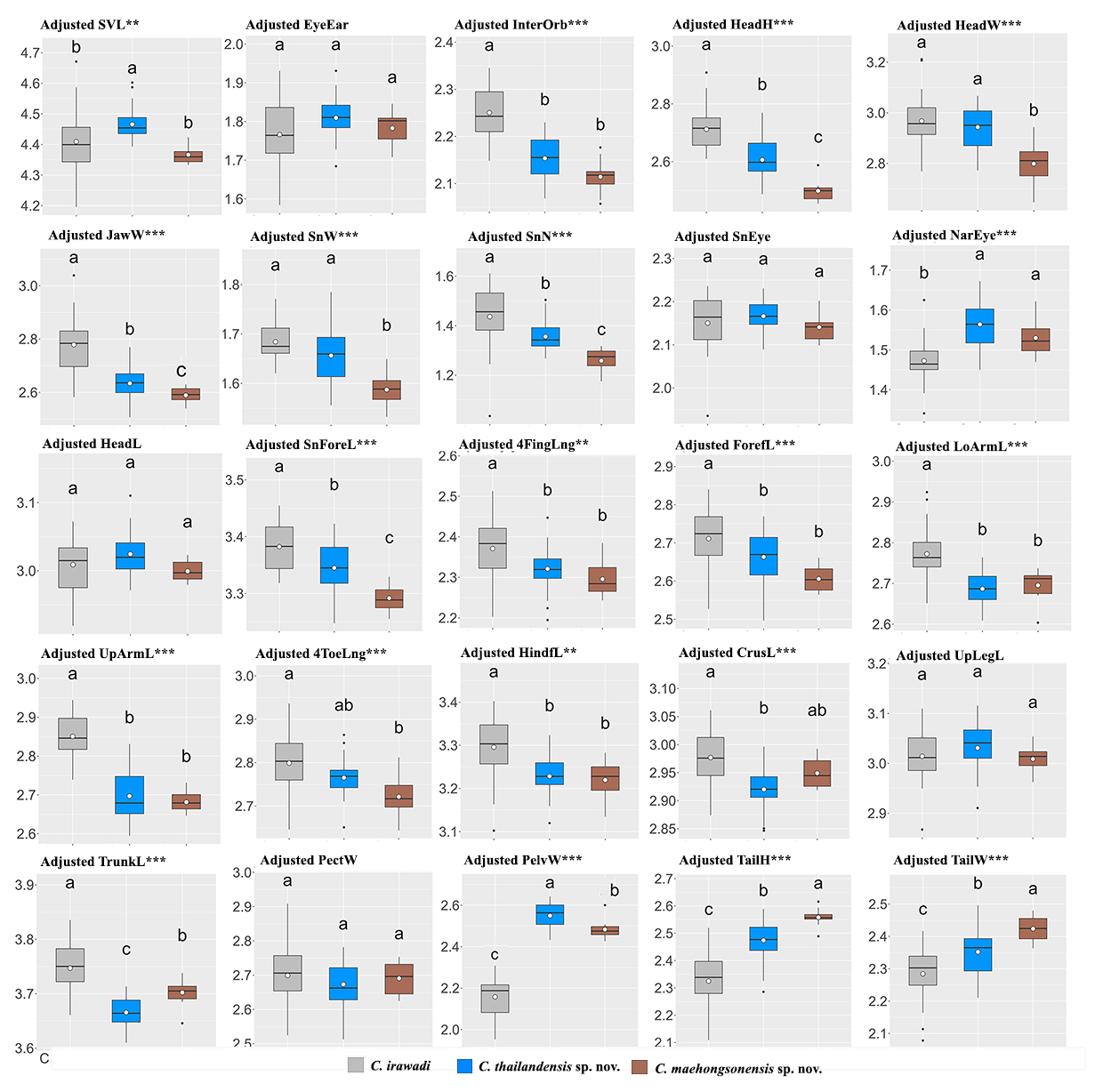

Supplement: Supplementary material 5 — Supplementary information 5 [file zookeys-1281-069_article-175455__-s005.zip › 175455_1C-1-A_supplementary_Figure_1.tif]

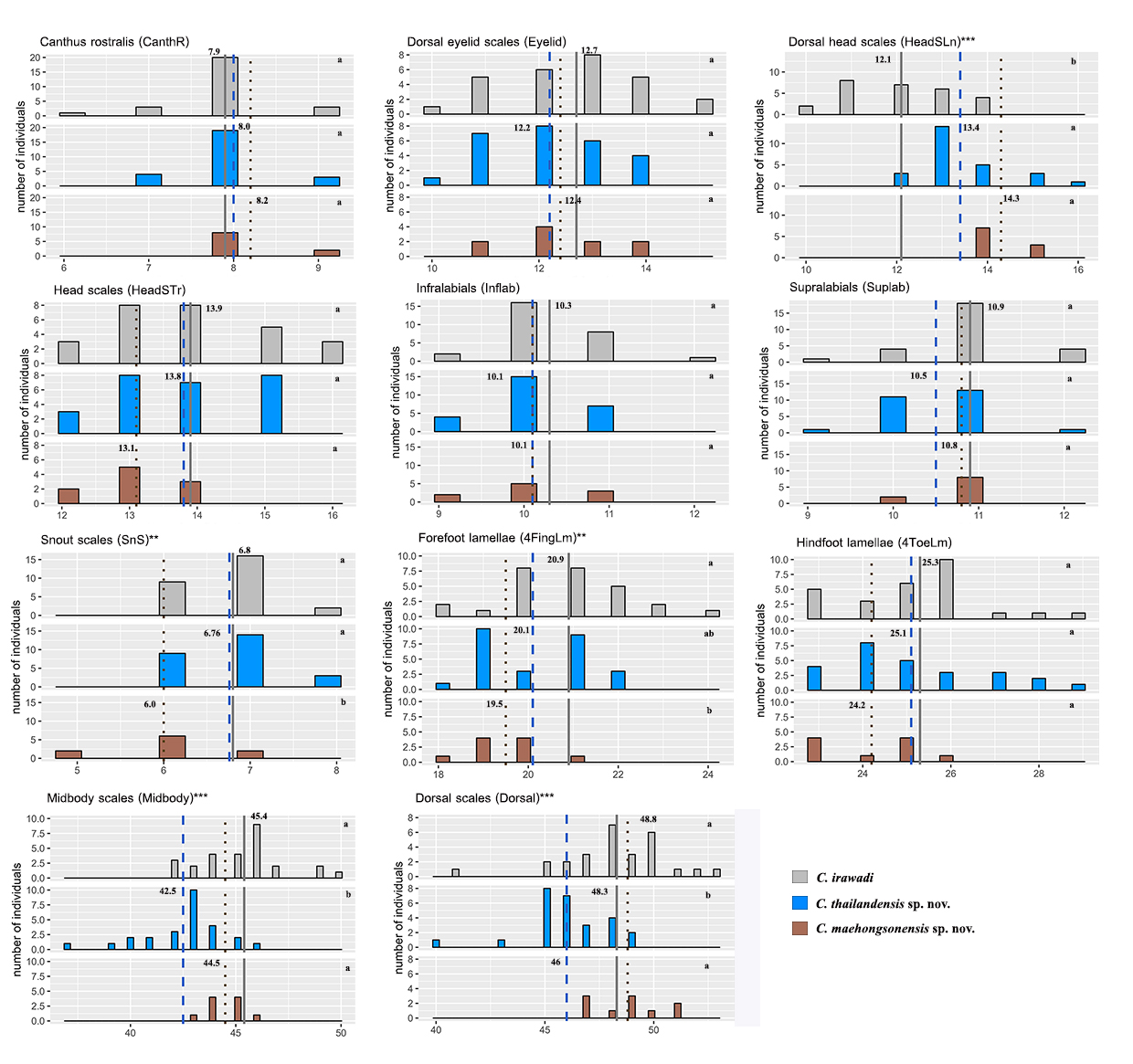

Supplement: Supplementary material 5 — Supplementary information 5 [file zookeys-1281-069_article-175455__-s005.zip › 175455_1C-1-A_supplementary_Figure_2.tif]

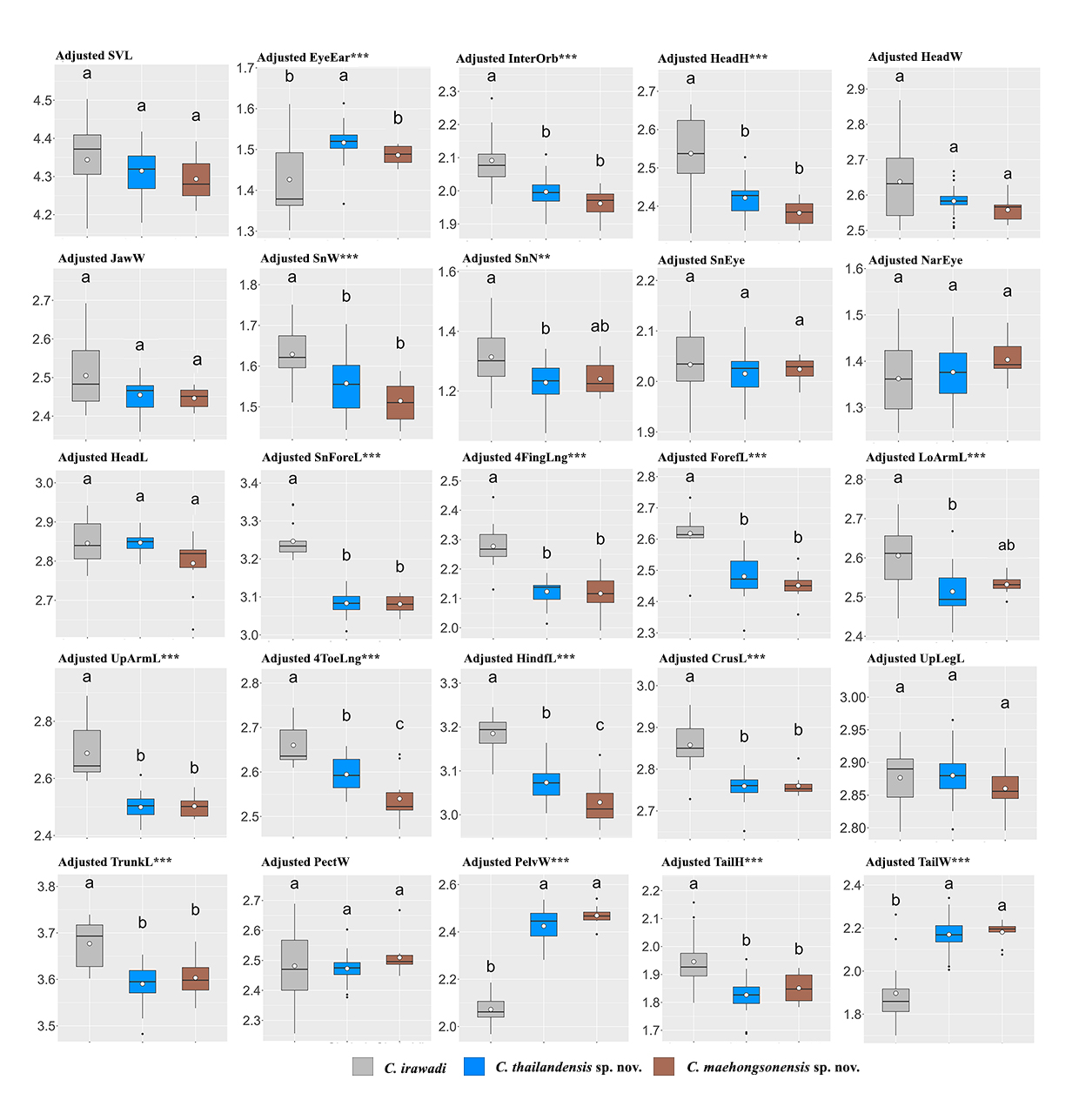

Supplement: Supplementary material 5 — Supplementary information 5 [file zookeys-1281-069_article-175455__-s005.zip › 175455_1C-1-A_supplementary_Figure_3.tif]
